# Xevinapant plus Chemoradiotherapy Negatively Sculpts the Tumor-Immune Microenvironment in Head and Neck Cancer

**DOI:** 10.1158/2767-9764.CRC-25-0604

**Published:** 2025-11-27

**Authors:** Charleen Chan Wah Hak, Emmanuel C. Patin, Anton Patrikeev, Annalisa Nicastri, Zuza Kozik, Holly Baldock, Joan N. Kyula-Currie, Victoria Roulstone, Amarin Wongariyapak, Valentina Gifford, Tencho Tenev, Elizabeth S. Appleton, Lisa C. Hubbard, Shane Foo, Malin Pedersen, Jyoti S. Choudhary, Masahiro Ono, Alan A. Melcher, Antonio Rullan, Kevin J. Harrington

**Affiliations:** 1 Division of Radiotherapy and Imaging, The Institute of Cancer Research, London, United Kingdom.; 2 Functional Proteomics Group, The Institute of Cancer Research, London, United Kingdom.; 3 Biological Services Unit, The Institute of Cancer Research, London, United Kingdom.; 4 The Breast Cancer Now Toby Robins Research Centre, The Institute of Cancer Research, London, United Kingdom.; 5 Department of Life Sciences, Faculty of Natural Sciences, Imperial College London, London, United Kingdom.

## Abstract

Xevinapant is an orally bioavailable antagonist of select members of the inhibitor of apoptosis protein family. Despite promising phase II data, combining xevinapant with chemoradiotherapy (CRT) failed to improve outcomes in the phase III TrilynX trial when combined with CRT for locally advanced head and neck squamous cell cancer (SCCHN). In immunocompetent mouse models of SCCHN, xevinapant plus CRT maintained or improved locoregional control but in a CD8^+^ T cell–independent manner. On addition of xevinapant to CRT, the numbers of tumor-infiltrating cytotoxic CD8^+^ T cells and NK cells were reduced, with remaining CD8^+^ T cells characterized by PD-1^hi^ CD38^hi^ expression and *Nr4a3* dynamics consistent with nonresponsiveness to antigenic restimulation. Furthermore, combination treatment significantly downregulated gene expression associated with immune-related pathways, increased levels of immunodysregulatory acute-phase proteins, and decreased levels of necroptosis mediator receptor-interacting protein kinase 3. Overall, xevinapant plus CRT has an immunosuppressive effect on the tumor-immune microenvironment, which may explain its lack of clinical benefit.

**Significance:**

Despite hugely promising randomized phase II study data, combined CRT plus xevinapant failed in the TrilynX phase III clinical trial in locally advanced SCCHN. We show that adding xevinapant to chemoradiotherapy *in vivo* dysregulates antitumor lymphocyte function, acute-phase proteins, and cell death pathways, with net immunosuppressive effects on the tumor-immune microenvironment.

## Introduction

The incidence of head and neck squamous cell cancer (SCCHN) has increased over the last decade by 16% (1). It is now the seventh most common cancer diagnosis worldwide (2). Most patients (60%) are diagnosed with locally advanced (LA) disease, defined by the Unio Internationale Contra Cancrum Tumor–Node–Metastasis eighth edition as either stage III or IV oral cavity, laryngeal, hypopharyngeal, and p16-negative oropharyngeal cancers or T3 to T4/N0 to N3 and T0 to T4/N1 to N3 p16-positive oropharyngeal cancer (3). The current treatment options available for patients with LA-SCCHN are either surgery followed by radiation or chemoradiotherapy (CRT) or definitive CRT for unresected disease (4–7).

The most widely used CRT regimens, irrespective of tumor location, consist of cisplatin 100 mg/m^2^ every 3 weeks or 40 mg/m^2^ weekly, combined with ∼70 Gy radiation delivered in 1.8 to 2.0 Gy daily fractions (7, 8). Although the addition of cisplatin is associated with increased toxicity compared with radiotherapy (RT) alone, this combination improves local control, disease-free/event-free survival, and overall survival (OS; ref. 8, 9). Nevertheless, despite these treatments, approximately half of patients with LA-SCCHN will develop local disease recurrence and/or metastatic disease within 2 years of completing treatment (10). The prognosis for these patients remains poor with a median OS of approximately 12 to 18 months despite treatment (11, 12). The recent success of KEYNOTE-689 in resectable LA-SCCHN, in which neoadjuvant and adjuvant pembrolizumab significantly improved event-free survival, highlights the promise of immune checkpoint blockade in the perioperative setting (13). In contrast, phase III clinical trials testing immune checkpoint inhibitors with definitive CRT for unresectable LA-SCCHN have, thus far, been disappointing (14–20), leaving the standard-of-care largely unchanged over the last two decades. This therapeutic divergence underscores the need for mechanistic preclinical studies, such as ours, to define how CRT and novel agents reshape the tumor-immune microenvironment and to guide rational development of effective immunomodulatory strategies for unresectable disease.

Inhibitor of apoptosis proteins (IAP) are a family of proteins that are overexpressed in numerous cancers, including SCCHN, and represent poor predictive (21) and prognostic factors (22–24). These proteins are named for their ability to regulate apoptosis. Among these, X chromosome–linked IAP is the only direct inhibitor of caspase-3, -7 and -9 via its baculovirus IAP repeat domains. Cellular IAP1 (cIAP1) and cIAP2 inhibit caspase-3 and -7 through their really interesting new gene finger domains which add polyubiquitin chains and target them for proteasomal degradation (25, 26). cIAP1 and cIAP2 are also involved in the regulation of death receptor–mediated apoptosis (27). In addition to their role in suppressing apoptosis, IAPs are increasingly implicated as modulators of NF-κB and associated downstream signaling pathways that affect cellular processes commonly dysregulated in human cancers, such as inflammation, innate and adaptive immunity, cell migration, and tumor cell survival (28, 29).

Xevinapant (Debio 1143, AT-406, and SM-406) is an orally bioavailable second mitochondria-derived activator of caspase mimetic or small-molecule antagonist of IAPs, including X chromosome–linked IAP, cIAP1, and cIAP2. *In vitro* and *in vivo* preclinical studies have shown that xevinapant sensitizes cancer cells to both chemotherapy (30, 31) and radiation (27, 32) and has immunomodulatory potential (33). Xevinapant exerted a radiosensitizing effect *in vitro* through modulation of caspases and TNFα in human head and neck cell lines, and this translated to a combination effect in xenograft models (27). Another study, in lung cancer, showed that xevinapant enhanced the efficacy of ablative RT *in vivo* in a TNFα-dependent, IFN-γ–dependent, and CD8^+^ T cell–dependent manner (33). A window of opportunity study in preoperative patients with SCCHN receiving xevinapant monotherapy showed that the mean intratumoral drug concentration was 18-fold greater than that in plasma, with significant engagement and degradation of cIAP1 (34). In addition, CD8^+^ tumor-infiltrating lymphocytes (TIL) and PD-1–positive and PD-L1–positive immune cells were significantly increased following xevinapant (34).

A phase II randomized, placebo-controlled trial showed superior locoregional control (54% vs. 33%) at 18 months with the addition of xevinapant versus placebo to standard-of-care CRT (35). At 3-year follow-up, OS and progression-free survival also showed significant benefit from xevinapant versus placebo, with an acceptable safety profile of xevinapant that did not jeopardize the delivery of standard-of-care CRT (36). Given the fact that this was the first treatment regimen to outperform standard-of-care CRT in a randomized trial, the registrational TrilynX study (NCT04459715) was set up in unresected LA-SCCHN and recruited rapidly. Disappointingly, however, a preplanned interim analysis concluded that TrilynX would not meet its primary endpoint of event-free survival (37), and the study was discontinued, as was the related phase III XRay Vision study (NCT05386550) of xevinapant plus RT compared with placebo plus RT in patients with resected LA-SCCHN at high risk of relapse (37). Critically, analysis of the data from the TrilynX study has revealed that locoregional control was equivalent in the CRT plus xevinapant and CRT plus placebo arms; however, distant metastasis was much more common in patients randomized to xevinapant (38). This finding raises the intriguing possibility that xevinapant may exert radiosensitizing effects in the irradiated field but, simultaneously, may have immunosuppressive effects predisposing to longer-term systemic failure.

We hypothesized that this class of agents may exert deleterious immunosuppressive effects that counteract their ability to sensitize to CRT. Our study aims to use preclinical immunocompetent models of head and neck cancer, treated with xevinapant in combination with CRT, to dissect these mechanisms—albeit providing hypothesis-generating insights rather than direct clinical extrapolations—to elucidate the effects of IAP inhibition on the tumor-immune microenvironment.

## Materials and Methods

### Tissue culture

The syngeneic C57BL/6 mouse cancer cell lines mEER (RRID: CVCL_B6J2) and MOC1 (RRID: CVCL_ZD32) were kindly provided by Paola Vermeer (Sanford Research) and Ravindra Uppaluri (Dana-Farber Cancer Institute), respectively. Cells were cultured and maintained in DMEM (Sigma-Aldrich, D6429) supplemented with 5% or 10% heat-inactivated FBS (Gibco, A5256801), 1% L-glutamine, and 0.5% penicillin/streptomycin. The cell lines used in the experiments were passaged for approximately 5 to 10 passages before a new aliquot was thawed. Cell line authentication was performed using whole-exome sequencing. *Mycoplasma* contamination was routinely monitored using the MycoStrip Mycoplasma Detection Kit (InvivoGen, RRID: SCR_026997), and no infection was detected.

### Cell viability assay

CellTiter-Glo cell viability assay (Promega, G7571) was performed according to the manufacturer’s instructions. Briefly, 5,000 mEER or MOC1 cells in 5% complete DMEM were plated in 96-well clear flat-bottom white plates (Greiner Bio-One, 655098) and left to adhere. After 24 hours, cells were treated with vehicle of 0.1% DMSO or various indicated treatments with a final concentration of xevinapant 10 μmol/L (Stratech Scientific Ltd., S2754), emricasan 5 μmol/L (MedKoo, 510230), or cisplatin 5 μmol/L (Accord, 16729-288-11), 30 minutes prior to 8 Gy irradiation using an AGO 250 kV X-ray machine (AGO, 20090606). At 48 hours following treatment, cell survival was measured using a SpectraMax iD5 plate reader (Molecular Devices).

### Cell death assay

A total of 5,000 mEER or MOC1 cells in 5% complete DMEM were plated in 96-well clear flat-bottom black plates (Greiner Bio-One, 655090) and left to adhere for 24 hours prior to indicated treatments. At 48 hours following treatment, final concentrations of Hoechst 0.5 μg/mL (Thermo Fisher Scientific, 33342) and propidium iodide 1 μg/mL (Sigma-Aldrich, P4170) were added and incubated at 37°C for 30 minutes. Following this, the percentage of dead cells was measured using the Celigo S cell imaging cytometer (Nexcelom Bioscience).

### Western blotting

mEER or MOC1 cells were plated at 1 × 10^6^ in Nunc p100 dishes (Thermo Fisher Scientific, 150350). After 24 hours, cells received indicated treatments and were collected 48 hours after treatment. Cells were washed in ice-cold PBS and pelleted by centrifugation at 4°C for 5 minutes at 2,400 rpm. Cells were then resuspended in 100 μL RIPA buffer (Thermo Fisher Scientific, 10230544) supplemented with protease inhibitors (Roche, 11836153001), 100 μL of NaF, and 50 μL of Na_3_VO_4_ to probe phosphoproteins. Cells were snap-frozen on dry ice and lysates subsequently allowed to thaw on ice before centrifugation at 4°C for 20 minutes at 13,200 rpm to remove cell debris. The protein concentration was determined using the bicinchoninic acid protein assay reagent (Pierce, 23225), and 30 μg of each protein sample was resolved on 10% NuPAGE Bis-Tris gels (Invitrogen, NP0302BOX) and transferred to a polyvinylidene difluoride Hybond-P membrane (Thermo Fisher Scientific, 88518). Antibodies for immunodetections were performed using caspase-3 (Cell Signaling Technology, 9665; RRID: AB_2069872) and PARP-1 (Santa Cruz Biotechnology, sc-8007; RRID: AB_628105). Equal loading was assessed using a β-actin antibody (Abcam, ab6276; RRID: AB_10920058). Blots were developed using antimouse (GE HealthCare, NA934; RRID: AB_772210) secondary antibody conjugated to horseradish peroxidase. Detection was performed using the SuperSignal West Pico chemiluminescent substrate (Pierce, 34580) or Immobilon (Millipore, WBKLS0500). Protein visualization was achieved using the Curix 60 image processor (Agfa) or the chemiluminescent Western blot imager (Azure 300).

### Mouse models of head and neck cancer

All procedures involving animals were approved by the Animal Ethics Committee at The Institute of Cancer Research, United Kingdom, in accordance with National Home Office Regulations under the Animals (Scientific Procedures) Act 1986. Eight- to 14-week-old female C57BL/6 wild-type (WT; Charles River Laboratories, RRID: MGI:2159769) or *Nr4a3*-Tocky: *Foxp3*-EGFP mice (39) were inoculated subcutaneously into the right flank with 1 × 10^6^ mEER or 4 × 10^6^ MOC1 cells suspended in 100 μL PBS under isoflurane anesthesia. Tumor and weight measurements were taken twice weekly using calipers by an independent animal technician. Tumor volume (mm^3^) was calculated using the formula length (mm) × width (mm) × height (mm) × 0.5236. For survival experiments, mice were sacrificed once 15 mm was reached in any tumor dimension, or when weight loss of ≥20% of baseline weight was observed. For immunophenotyping or sequencing experiments, tumors were harvested at specified timepoints after treatment.

### *In vivo* tumor treatments

For all studies, mice were randomized prior to therapy for comparable tumor volumes between cohorts. *In vivo* treatments were commenced when tumor volumes reached approximately 20 to 50 mm^3^. Mice were immobilized for irradiation via a single injection of ketamine 100 mg/kg (Ketavet) and xylazine 16 mg/kg (Rompun) administered intraperitoneally. Mice were irradiated in the prone position under lead shielding with a 15-mm-diameter aperture aligned over the tumor. A total of 6 or 8 Gy × 3 fractions on alternate days were delivered at a dose rate of 1.62 Gy/minute using an AGO 250 kV X-ray machine (AGO, 20090606) with dose measurements performed with a Farmer chamber and UNIDOS E dosemeter (PTW). Following irradiation, cages were placed on heat mats, and mice were monitored until full recovery. Xevinapant powder for *in vivo* treatments was provided by the healthcare business of Merck KGaA and dissolved in vehicle formulated with 0.6% malic acid (Sigma-Aldrich, 240176; w/v) in 85 mmol/L sodium acetate buffer (Calbiochem, 7610), pH 4.5. Xevinapant was administered at 100 mg/kg via oral gavage administered for five consecutive days followed by 2 days off, repeated twice over the course of 3 weeks. Cisplatin powder (Sigma-Aldrich, P4394) was diluted in 0.9% sodium chloride on the day of use and 5 to 7 mg/kg was delivered by intraperitoneal injection 1 hour prior to radiotherapy (if given). For depletion of CD8^+^ T cells, a loading dose of 400 μg/mouse of anti-CD8α (Bio X Cell, clone: 2.43, BE0061; RRID: AB_1125541) or rat IgG2b isotype (Bio X Cell, clone LTF-2, BE0090; RRID: AB_3662740) was administered 24 hours prior to treatment initiation, followed by maintenance doses twice weekly for up to 10 doses.

### RNA sequencing

Mouse tumors were harvested 14 days after the last radiation dose, on the last day of vehicle/xevinapant treatment, and snap-frozen on dry ice. Tumor tissue was homogenized in 1 mL of Buffer RLT Plus (Qiagen, 1053393) with 10 μL β-mercaptoethanol (Gibco, 21985023) within CK28 hard tissue homogenizing 2-mL tubes (Precellys, 10144-494) using the Precellys Evolution homogenizer. RNA from tumors was extracted and purified from tumors using the RNeasy Plus Mini Kit (Qiagen, 74134) with QIAshredder (Qiagen, 79656) according to the manufacturer’s protocol and stored at −80°C. Library preparation and sequencing were performed by GENEWIZ, Germany. Library preparation was strand-specific with polyA selection, with 2 × 150 bp sequencing at 20 million paired-end reads per sample performed on an Illumina NovaSeq. Salmon quant v1.10.1 (40) was used for transcript-level quantification against the GRCm39 mouse reference (Ensembl release 103). Transcript-level abundance estimates were aggregated to the gene level using the tximport v1.34 R package (41). Differential gene expression analysis was performed using DESeq2 (RRID: SCR_000154; pseudogenes were excluded, and only genes with more than 10 reads in at least given samples were kept in the analysis; ref. 42). Cell-type signature genes were obtained from single-cell RNA sequencing data from patients with oropharyngeal squamous cell carcinoma (43), and signature scores were calculated using the Gene Set Variation Analysis (GSVA) package (v2.0.4; ref. 44) applied to counts normalized using variance-stabilizing transformation. Gene set enrichment analysis was performed using the clusterProfiler package (v4.14.4; RRID: SCR_016884; ref. 45).

### Flow cytometry

Mouse samples collected for flow cytometry included tumor and tumor-draining lymph nodes in PBS on ice. Tumors were weighed at the time of acquisition and dissociated mechanically and enzymatically digested in PBS containing 0.5 mg/mL collagenase type 76 I-S (Sigma-Aldrich, C1639), 0.4 mg/mL Dispase II protease (Sigma-Aldrich, D4693), 0.2 mg/mL DNase I (Roche, 10104159001), and 4% trypsin (0.25% in Tris-saline) for 30 minutes at 37°C on a shaker for WT samples or 10 minutes at 37°C followed by 20 minutes at room temperature for Timer of Cell Kinetics and Activity (Tocky) samples. Digested tumor samples and undigested lymph nodes were passed through a 70-μm cell strainer (Fisherbrand, 11597522) and washed with FACS buffer consisting of 2% FBS in PBS, supplemented with 5 mmol/L ethylenediaminetetraacetic acid. Cells were centrifuged at 300 × *g* at 4°C for 5 minutes and incubated with anti–mouse CD16/CD32 Fc-receptor blocker [Becton Dickinson, 553142] at 4°C for 10 minutes prior to extracellular staining. Dead cells were excluded using the eBioscience Fixable Viability Dye eFluor 780 (Thermo Fisher Scientific, 65-0865-14). For intracellular staining, the Foxp3/Transcription Factor Staining Buffer Set (eBioscience, 00-5523-00) was used to permeabilize and fix cells according to the manufacturer’s protocol prior to intracellular staining. A complete list of antibodies used in this study can be found in Supplementary Data. Samples were then resuspended in FACS buffer and stored at 4°C in the dark until acquisition on the BD FACSymphony A5 cell analyzer (BD Biosciences) within a week of fixation for WT samples or within the same day for unfixed Tocky samples. CountBright absolute counting beads 25 to 50 μL (Invitrogen, C36950) were run with tumor samples. Compensation was performed using either single-stained lymph node cells or Ultracomp eBeads compensation beads (Invitrogen, 01-2222-42). The gating strategy is detailed in Supplementary Data (Supplementary Fig. S7). FACS analyses were performed using FlowJo version 10 software (RRID: SCR_008520), Tocky data were analyzed using the TockyAnalysis package in R v. 3.6.3, and Uniform Manifold Approximation and Projection for Dimension Reduction (UMAP) analyses (bioRxiv 2022.07.19.500582) were performed using R v. 4.3.2.

### IHC

Mouse specimens (tumor or lung) were fixed in 10% neutral-buffered formalin (Sigma-Aldrich, HT501128) for 24 hours at room temperature, after which they were transferred to PBS at 4°C. Paraffin embedding of fixed material and IHC staining were performed by The Institute of Cancer Research Histopathology Core Facility. Anti-CD45 (eBioscience, clone: 30-F11, 14-0451-82; RRID: AB_10701459) was used with heat-induced epitope retrieval using Agilent Target Retrieval solution pH 6 (K8005) and Nichirei anti-rat N-Histofine reagent (414311F) as the detection system. Anti-CD8a (Abcam, clone: EPR21769, ab217344, RRID: AB_2890649; eBioscience, clone: 4SM15, 14-0808, RRID: AB_2572861) was used for heat-induced epitope retrieval using Agilent Target Retrieval solution pH 9 (K8004) and Agilent rabbit EnVision reagent (K4003) as the detection system.

### Proteomics

Mouse tumors were harvested 14 days after the last radiation dose, on the last day of vehicle/xevinapant oral gavage, and snap-frozen in liquid nitrogen immediately after collection. When ready for processing, murine tumors were thawed on ice and 1 mL of lysis buffer was added, containing 100 mmol/L triethylammonium bicarbonate, 1% sodium deoxycholate, 10% isopropanol, 5 mmol/L tris(2-carboxyethyl)phosphine, 10 mmol/L iodoacetamide, 50 mmol/L NaCl, and a Halt protease and phosphatase inhibitor cocktail (100×). Tissue homogenization was performed on ice using probe sonication. The protein concentration was determined using the Quick Start Bradford assay following the manufacturer’s instructions. For whole-proteome analysis, 25 μg of protein per sample was digested overnight at 37°C with trypsin. Peptides were labeled with TMT10plex reagents before pooling all 10 samples and acidified with 1% formic acid, and the precipitated sodium deoxycholate was removed by centrifugation at 10,000 rpm for 5 minutes at room temperature and finally dried using SpeedVac. TMTpro-labeled peptides were fractionated using high-pH reversed-phase chromatography on an XBridge C18 column (2.1 × 150 mm, 3.5 μm, Waters) with a Dionex UltiMate 3000 high-performance liquid chromatography system at a flow rate of 0.2 mL/minute. The gradient program was started at 5% buffer B for 5 minutes, then 12% B over 3 minutes, a gradual increase to 35% B over 32 minutes, and then increased to 80% B within 5 minutes, followed by re-equilibration at 5% B. Fractions were collected every 42 seconds and dried using SpeedVac. Peptides were resuspended in 0.1% trifluoroacetic acid and loaded onto the Acclaim PepMap 100, 100 μm × 2 cm C18, 5 μm, trapping column at a flow rate 10 μL/minute and analyzed using an Acclaim PepMap (75 μm × 50 cm, 2 μm, 100 Å) C18 capillary column connected to a stainless steel emitter on an EASY-Spray source via a PSS2 adapter (MS Wil). A 100-minute gradient from 5% to 35% buffer B was used. Mass spectrometry scans were acquired in the m/z range of 400 to 1,600 with a mass resolution of 120K, a standard automatic gain control target, and a maximum injection time of 35 ms. Precursors were selected using a top-speed mode with 3-second cycles and isolated for higher-energy collisional dissociation (HCD) fragmentation with a quadrupole isolation width of 0.7 Th. Fragmentation was performed at a collision energy of 32%. Quantification was obtained at the MS3 level with HCD fragmentation of the top 10 most abundant collision-induced dissociation fragments isolated with synchronous precursor selection. Quadrupole isolation width was 0.7 Th, collision energy was 55%, and automatic gain control AGC was set to 1 × 10^5^ with 200 ms max IT. The HCD MS3 spectra were acquired for the mass range of 100 to 500 m/z with 45K resolution. Targeted precursors were dynamically excluded from further fragmentation for 30 seconds with a 25-ppm mass tolerance. Mass spectra were analyzed using Proteome Discoverer 3.0 (Thermo Fisher Scientific; RRID: SCR_014477) with the Sequest HT search engine for peptide identification and quantification. Precursor and fragment ion mass tolerances were set to 20 ppm and 0.02 Da, respectively. The analysis parameters included fully tryptic peptides with up to two missed cleavages, TMTpro modifications as static at the N-terminus and lysine residues, and carbamidomethylation as a static modification at cysteine residues. Peptide searches were conducted against reviewed UniProt (RRID: SCR_002380) *Mus musculus* protein entries, with confidence assessed using the percolator node. Peptides were filtered based on a target-decoy database search, applying a *q* value threshold of <0.01. The reporter ion quantifier node employed a TMTpro quantification method with an integration window tolerance of 15 ppm. Only peptides with an average reporter signal-to-noise ratio >3 were considered for quantification. Differential protein expression analyses were performed using R version 4.4.2.

### Statistical methods

Statistical analyses were performed using Prism v10 (GraphPad; RRID: SCR_002798). Data were presented as mean ± SEM. AUC was used to determine statistical differences in tumor growth. Survival and time-to-event analyses were performed using the log-rank Mantel–Cox test. Flow cytometry marker expression was compared using an unpaired *t* test or one-way ANOVA followed by a *post hoc* Tukey test when two or multiple groups were analyzed, respectively. If data did not adhere to a Gaussian distribution, a nonparametric Mann–Whitney U or Kruskal–Wallis test followed by the Dunn *post hoc t* test was used for statistical analysis when two or multiple groups were analyzed, respectively. Outliers were removed with the ROUT test at Q = 1%. Statistical significance was indicated either by numerical *P* values or with *, *P* < 0.05; **, *P* < 0.005; ***, *P* < 0.001, and ****, *P* < 0.0001.

## Results

### Locoregional control is maintained or improved by xevinapant plus CRT independent of CD8^+^ T cells

**Figure 1.**
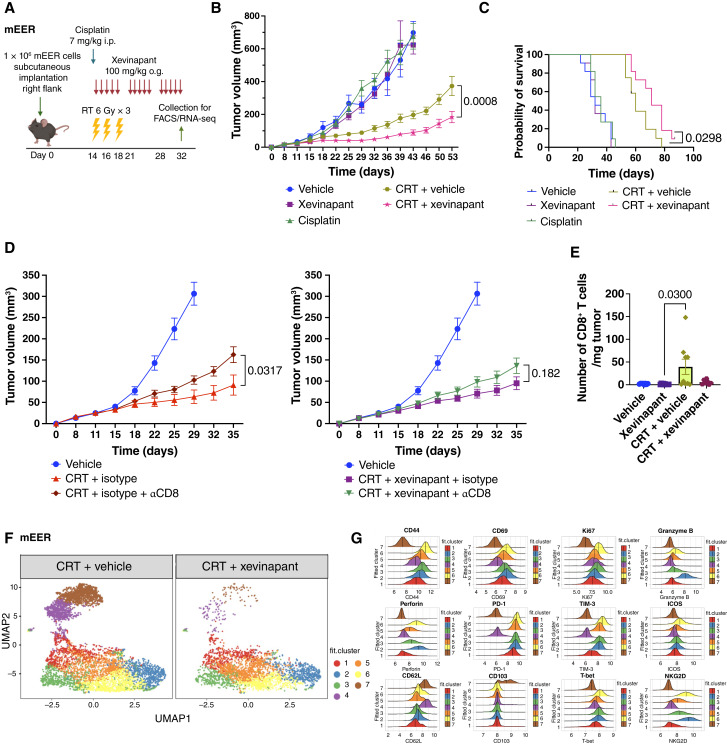
Locoregional control was maintained or improved with the combination of xevinapant and CRT *in vivo* in a CD8^+^ T cell–independent manner. Data presented in this figure were obtained using the mEER model. **A,** Treatment schematic for mEER. C57BL/6 mice were implanted with mEER cells (1 × 10^6^) subcutaneously in the right flank. Treatment included cisplatin (7 mg/kg i.p.), RT (6 Gy × 3 on alternate days), and xevinapant [100 mg/kg, oral gavage (o.g.), 5 consecutive days per week for 3 weeks]. Mice were monitored for tumor growth and survival or collected for downstream immunoprofiling (11–12 mice/group). **B,** Average tumor growth curves across different treatment conditions. **C,** Kaplan–Meier survival curves across different treatment conditions. **D,** Tumor growth curves of *in vivo* anti-CD8 (αCD8) depletion (11–15 mice/treatment group). αCD8 depleting antibody and isotype control were administered with a loading dose of 400 μg i.p. 24 hours prior to the start of treatment followed by a maintenance dose of 200 μg i.p. twice weekly for a total of 10 doses. **E,** Quantification of the number of CD8^+^ T cells per mg tumor using flow cytometry under indicated treatment conditions (8–10 mice/group). **F,** UMAP plot of CD8^+^ TILs comparing clusters 1–7 in CRT plus vehicle vs. CRT plus xevinapant treatment groups. **G,** Histograms illustrate the indicated marker expression for each cluster, with results concatenated from 30 mice. All data are representative of ≥2 independent experiments. RNA-seq, RNA sequencing. [**A,** Created with Biorender. (2025) https://BioRender.com/gw61ewu.]

We studied the therapeutic effect of xevinapant in combination with CRT in two immunocompetent mouse models of SCCHN: mEER and MOC1. Cell-intrinsic viability experiments *in vitro* showed that tumor cell death, via apoptosis, was maximized with the addition of xevinapant to CRT (Supplementary Fig. S1). *In vivo*, mice bearing subcutaneous flank tumors received xevinapant via oral gavage over 3 weeks concurrently with and after CRT. In the mEER model (Fig. 1A), the addition of xevinapant to CRT significantly delayed tumor growth (Fig. 1B; Supplementary Fig. S2A), with a nonsignificant trend toward improved tumor control with xevinapant and RT alone (Supplementary Fig. S2B). Combining xevinapant with either RT or CRT led to significantly improved survival compared with both RT and CRT alone, respectively (Fig. 1C; Supplementary Fig. S2C). We also tested the combination treatment in the MOC1 model, which showed no significant difference in tumor control or survival (Supplementary Fig. S3A–S3E). In both models, in keeping with the clinical data from TrilynX, there was no detriment to locoregional control with the combination of CRT and xevinapant versus CRT alone.

Interestingly, in mEER, the tumor control exerted by CRT alone was partially CD8^+^ T cell–dependent, as CD8 depletion led to a significant loss of tumor control. However, CD8^+^ T-cell depletion did not significantly modify tumor growth in the xevinapant combination treatment group (Fig. 1D; Supplementary Fig. S2D). This observation suggested that the addition of xevinapant to CRT reversed the CD8^+^ T-cell dependency of tumor control either by reducing the numbers of CD8^+^ TILs (Fig. 1E) or by rendering the remaining CD8^+^ TILs ineffectual.

To further investigate these findings *in vivo*, we performed multiparameter flow cytometry analyses of TILs across the different treatment conditions. In the mEER CD8^+^ T-cell population, UMAP analyses showed that the addition of xevinapant to CRT selectively depleted clusters 4 (purple) and 7 (brown; Fig. 1F), which corresponded to CD44^lo^, CD69^lo^, Ki67^lo^, GrzmB^lo^, perforin^lo^, PD-1^lo^, TIM-3^lo^, T-bet^lo^, NKG2D^lo^, and CD62L^hi^ expression (Fig. 1G). Although there was depletion of these less activated and cytotoxic CD8^+^ T-cell subsets, combination treatment did not promote any compensatory expansion of other, more activated, cytotoxic clusters, which warranted further analysis.

### Xevinapant plus CRT reduces the numbers of cytotoxic CD8^+^ T cells and NK cells; residual CD8^+^ T cells exhibit a dysfunctional phenotype

**Figure 2.**
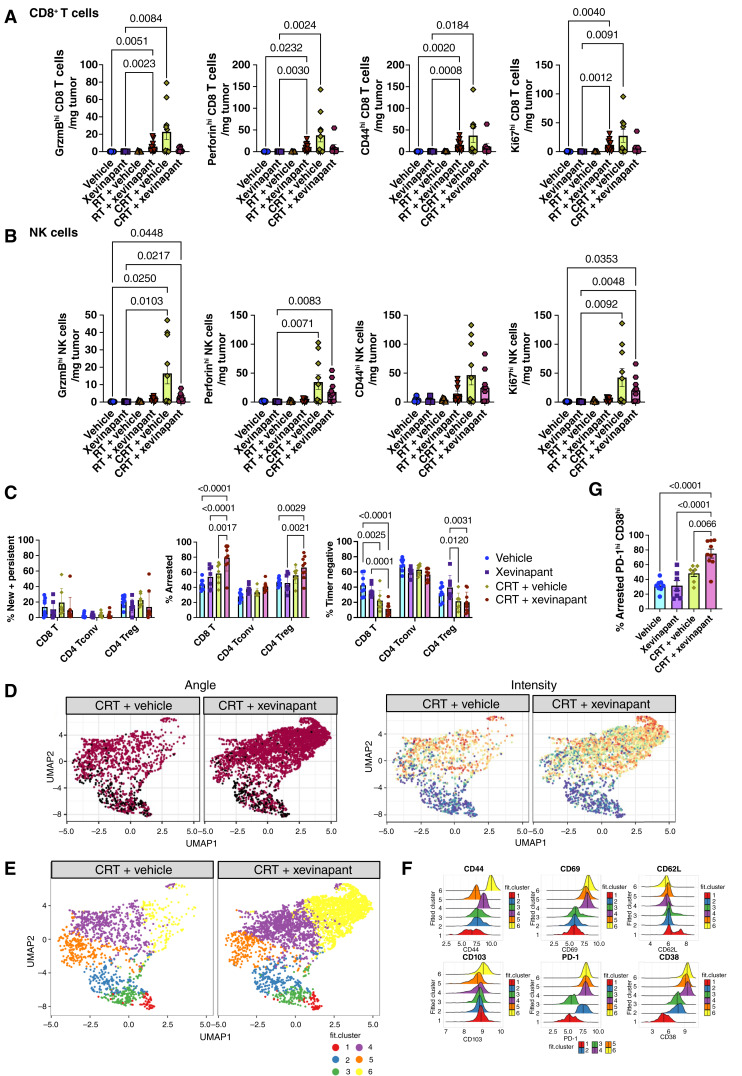
Xevinapant plus CRT reduced the numbers of cytotoxic CD8^+^ T cells and NK cells vs. CRT alone; remaining CD8^+^ T cells are PD-1^hi^ CD38^hi^ and not persistently engaged with antigens. Flow cytometry results presented in this figure were performed in the mEER model with WT mice (8–10/treatment group; **A** and **B**) and *Nr4a3*-Tocky mice (7–9/treatment group; **C–F**). **A,** Absolute counts of CD8^+^ T cells per mg of tumor are shown which express markers GrzmB^hi^, perforin^hi^, CD44^hi^, and Ki67^hi^. GrzmB, granzyme B. **B,** Absolute counts of NK cells per mg of tumor are shown which express markers GrzmB^hi^, perforin^hi^, CD44^hi^, and Ki67^hi^. **C,** The *Nr4a3*-Tocky system was used to analyze tumor-infiltrating CD8^+^, CD4^+^ conventional T (Tconv) cells, and CD4^+^ regulatory T cells (Treg). Representative dot plots show percentages of different fluorescent timer (FT) populations: combined “new” (FT blue+ FT red−) and “persistent” (FT blue+ FT red+); “arrested” (FT blue− FT red+) and “timer negative” (FT blue− FT red−). **D,** UMAP analyses of CD8^+^ TILs with overlay of Tocky parameters: “angle” (marker of antigen engagement) and “intensity” (marker of strength of TCR signaling). **E,** UMAP analyses of CD8^+^ T cells comparing clusters 1–6 between CRT plus vehicle vs. CRT plus xevinapant treatment groups. **F,** Histograms illustrate the indicated marker expression for each cluster, with results concatenated from 21 mice. **G,** Percentage of CD8^+^ T cells which are “arrested” (FT blue− FT red+), PD-1^hi^, and CD38^hi^ across different treatment conditions. All data are representative of two independent experiments.

In our responsive model, mEER, we found that the addition of xevinapant to CRT altered the activation, proliferation, and cytotoxicity of intratumoral lymphocytes in the tumor-immune microenvironment. Specifically, using multiparameter flow cytometry, we observed a trend toward reduced absolute numbers of intratumoral CD8^+^ T cells (Fig. 2A) and NK cells (Fig. 2B) expressing the degranulation markers granzyme B and perforin in the combination treatment versus CRT alone. A similar trend was also observed in the numbers of CD8^+^ T cells and NK cells expressing high levels of the activation and proliferation markers CD44 and Ki67, respectively. Notably, CRT alone, but not CRT plus xevinapant, led to significantly increased cell numbers expressing high levels of these cytotoxic and activation markers when compared with vehicle/xevinapant controls.

To study the kinetics of T-cell receptor (TCR) engagement, we used the “Tocky” *Nr4a3*-Tocky transgenic mouse reporter system (Supplementary Fig. S4; ref. 39). Within the mEER CD8^+^ T-cell population, a significantly higher percentage of “arrested” T cells was observed in the group receiving combination treatment than in the group receiving CRT alone (Fig. 2C). With UMAP analyses, we saw an increase in CD8^+^ “arrested” T cells with combination treatment that have historic antigen recognition (timer angle positive) and TCR signaling (timer intensity) but which are not continuously/persistently reengaging their TCR with cognate antigen (Fig. 2D). Phenotypically, these “arrested” CD8^+^ T cells in cluster 6 (yellow) express CD44^hi^, CD69^hi^, PD-1^hi^, and CD38^hi^ (Fig. 2E and F), and combination treatment led to significantly increased “arrested” PD-1^hi^ CD38^hi^ CD8^+^ T cells versus CRT alone (Fig. 2G). Interestingly, CD8^+^ T cells that co-express PD-1^hi^ and CD38^hi^ have been identified as suboptimally primed cells that signal erroneously through their TCR, display unresponsiveness to antigenic restimulation, and demonstrate resistance to anti–PD-1 (αPD-1) therapy (46). This may explain the accumulation in the “arrested,” rather than “persistent,” CD8^+^ T-cell antigen-engagement state with the combination treatment. To support our theory that the therapeutic efficacy of CRT plus xevinapant was not T cell–mediated, we tested the addition of αPD-1 to the combination treatment and found no significant improvement in tumor control or survival (Supplementary Fig. S2E).

### Immune-related gene expression is downregulated with xevinapant plus CRT versus CRT alone

**Figure 3.**
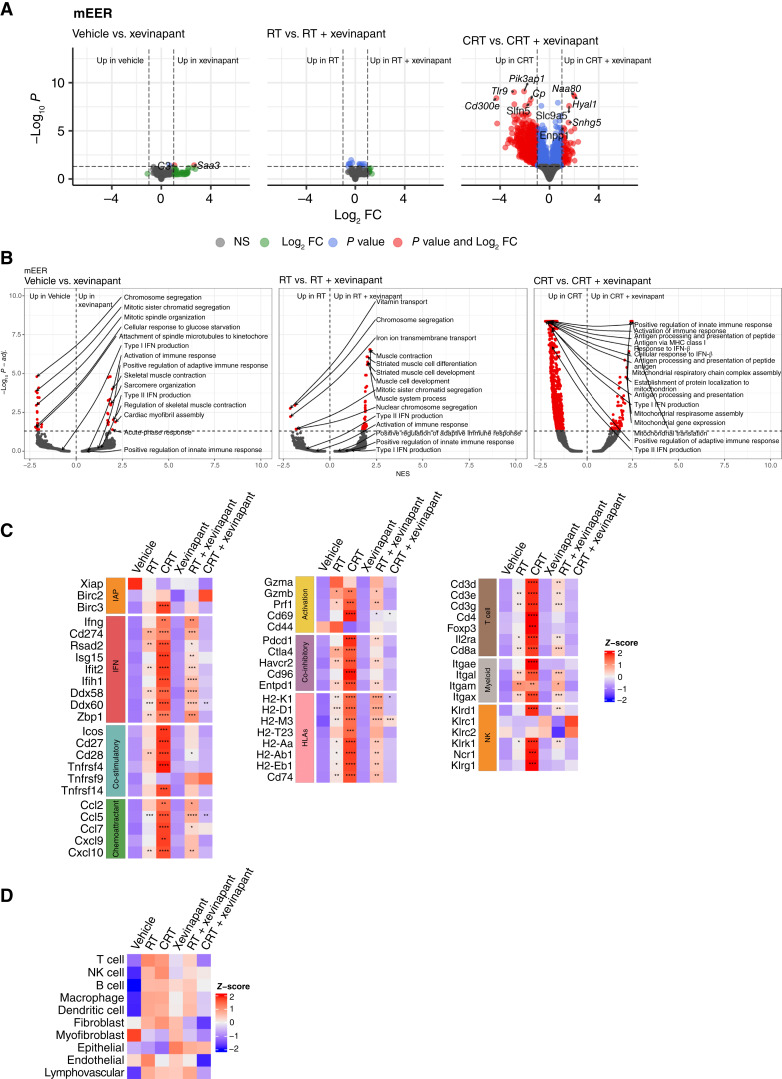
Gene expression associated with immune-related pathways is downregulated with xevinapant plus CRT vs. CRT alone. Data presented in this figure were performed in the mEER model and treatment regimen as described previously. **A,** Volcano plots showing differential expression of genes across the different treatment conditions; significant difference in gene expression was seen between CRT plus vehicle and CRT plus xevinapant groups. FC, fold change; NS, not statistically significant. **B,** Volcano plots for gene set enrichment analysis results (Gene Ontology biological process terms) across different treatment conditions. Pathways denoted in red represent terms with adjusted *P* values < 0.05 (corrected for multiple testing). Labels indicate the top five significantly up- and downregulated terms, as well as five immune-related processes of interest: type I/II IFN production, activation of immune response, and positive regulation of adaptive/innate immune response. NES, normalized enrichment score. **C,** Heatmaps corresponding to IAP, IFN, and cytokine signaling, co-stimulatory and chemoattractant pathways, activation status, immune checkpoint markers, human leukocyte antigens (HLA), and immune cell populations across different treatment conditions. Birc, baculoviral IAP repeat–containing protein; Grzma, granzyme A; Grzmb, granzyme B; Xiap, X chromosome–linked IAP. **D,** TME cell-type signature scores across different treatment conditions shown as *Z*-scores of mean GSVA signature scores per condition (5 tumors/group).

RNA sequencing analysis in the mEER model showed that the addition of xevinapant to CRT led to generalized immunosuppression of the tumor microenvironment (TME). Significant differences in gene expression were observed between CRT plus vehicle and CRT plus xevinapant treatment groups (Fig. 3A). CRT plus vehicle was shown to upregulate pathways related to innate and adaptive immunity relative to control (Fig. 3B). Specifically, there was significantly increased gene expression related to IFN and cytokine signaling, co-stimulatory and chemoattractant pathways, activation, immune checkpoints, human leukocyte antigens, and immune cell populations (Fig. 3C). In contrast, the addition of xevinapant to CRT profoundly reversed this effect across all the aforementioned pathways, resulting in CRT plus xevinapant having a similar immune profile to vehicle/xevinapant controls alone (Fig. 3C). Immune cell population estimates also showed that CRT plus vehicle, relative to control, enhanced T-cell, NK-cell, B-cell, macrophage, and dendritic cell numbers. However, the addition of xevinapant to CRT reduced the levels of these immune cell populations versus CRT plus vehicle (Fig. 3D). In the MOC1 model, although the addition of xevinapant to CRT did not have as marked a pan-immunosuppressive effect as in the mEER model, its overall effect can be characterized as immunosuppressive, particularly in the NK and T-cell compartments (Supplementary Fig. S5). Our data were further compared with two published gene signatures predicting immunotherapy responsiveness and prognosis in human SCCHN (47, 48), which notably feature many genes overlapping with those analyzed in our study. This revealed a similarly adverse pattern of signature gene downregulation following the addition of xevinapant to CRT, more pronounced in the mEER model than the MOC1 model (Supplementary Fig. S6).

### Xevinapant plus CRT favors apoptosis over necroptosis and increases expression of immunosuppressive acute-phase proteins

**Figure 4.**
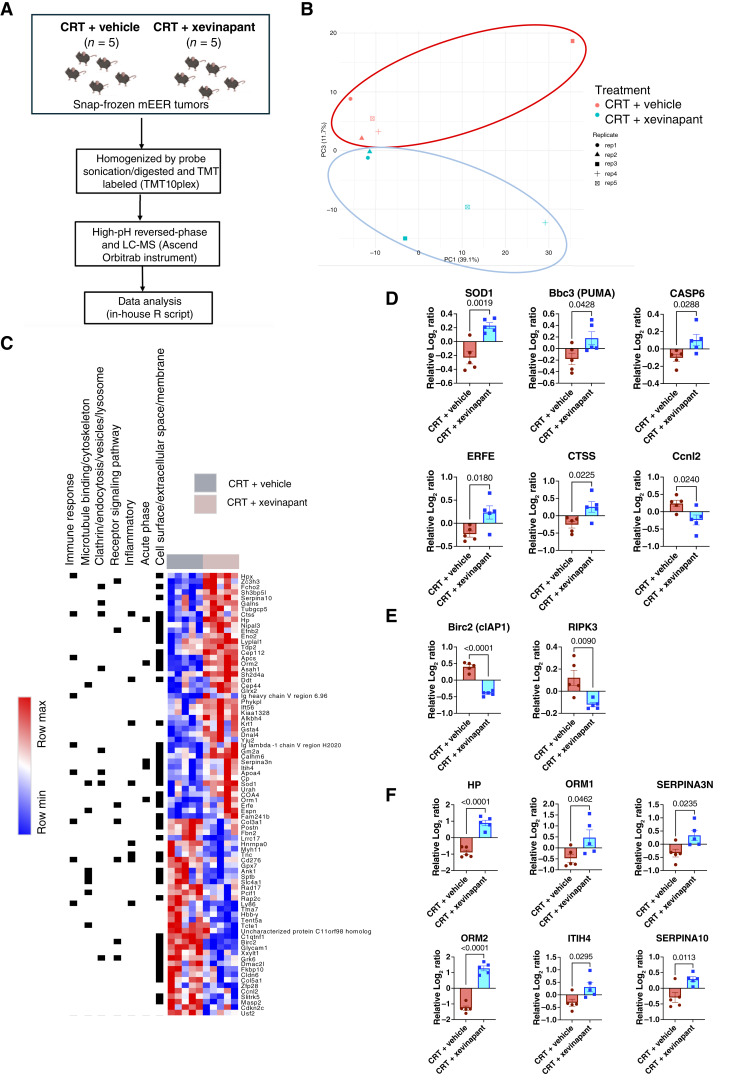
Xevinapant plus CRT significantly increased the expression of immunosuppressive acute-phase proteins and enhanced apoptotic rather than necroptotic cell death. Data presented in this figure were performed in the mEER model and treatment regimen as described previously. **A,** Whole-proteome analysis experimental design and methodology overview comparing CRT plus vehicle vs. CRT plus xevinapant. Proteomic analysis yielded a total of 7,288 proteins (5 tumors/group). **B,** Principal component analysis of the whole proteome. Principal component analysis plot of PC1 vs. PC3 shows separation between both treatment groups. PC, principal component. **C,** Proteome signature of significantly deregulated proteins (*n* = 81, *P* < 0.05, and log_2_ fold change > |0.38| between treatment conditions. Proteins significantly upregulated (*n* = 46) and downregulated (*n* = 35) illustrated via heatmap. **D,** Significant changes in the expression of proteins involved in the apoptotic pathway: superoxide dismutase 1 (SOD1), Bcl-2–binding component 3 (Bbc3/PUMA, encoded by *Bbc3*), caspase-6 (CASP6), erythroferrone (ERFE), cathepsin S (CTSS), and cyclin L2 (Ccnl2, encoded by *Ccnl2*). **E,** Inhibition of IAP is seen with a significant reduction in cell death protein baculoviral IAP repeat-containing protein 2 (Birc2/cIAP1, encoded by *Birc2*) and a significant reduction in RIPK3. **F,** Changes in the expression of acute-phase proteins: haptoglobin (HP), orosomucoid 1 (ORM1), serpin family A member 3 (SERPINA3N), orosomucoid 2 (ORM2), inter-alpha-trypsin inhibitor heavy chain 4 (ITIH4), and serpin family A member 10 (SERPINA10).

Whole-proteome analysis *in vivo* in the mEER model showed significant differences with respect to protein expression after treatment with CRT plus vehicle versus CRT plus xevinapant (Fig. 4A–C). Notably, several apoptotic pathway proteins, namely, superoxide dismutase 1, Bcl-2–binding component 3 (or PUMA, encoded by *Bbc3*), caspase-6, erythroferrone, and cathepsin S, were significantly upregulated in the combination group (Fig. 4D). The significant reduction in baculoviral IAP repeat–containing protein 2 was consistent with the expected inhibition of IAPs by xevinapant. However, there was a significant reduction in receptor-interacting protein kinase 3 (*Ripk3*) gene expression (log_2_ fold change = −0.42; *P* = 0.04) and protein level expression (RIPK3) with the addition of xevinapant to CRT versus CRT plus vehicle (Fig. 4E). RIPK3 reduction suggested that immunogenic necroptosis was reduced in the setting of combination treatment in favor of relatively immunogenically silent apoptosis. Furthermore, the addition of xevinapant to CRT significantly increased the expression of acute-phase proteins, namely, haptoglobin (49), orosomucoid 1 (50), and serpin family A member 3 (51), all of which reportedly have inhibitory effects on antitumor lymphocyte function (Fig. 4F).

Overall, we showed that xevinapant functions as a chemo/radiosensitizer for apoptotic cell death within the irradiated tumor of our responsive model mEER. The addition of xevinapant to CRT, however, leads to a wider immunosuppressive effect on the TME, which is detrimental to long-term adaptive antitumor immunity.

## Discussion

The phase III clinical trial, TrilynX, comparing CRT plus xevinapant versus CRT plus placebo in unresected LA-SCCHN did not meet its primary endpoint of event-free survival and was terminated following interim analysis. Results showed that although locoregional control was equivalent in the CRT plus xevinapant and CRT plus placebo arms, distant failure was more common in patients randomized to xevinapant (38). In keeping with the clinical effect of xevinapant, our study has found no detriment of CRT plus xevinapant versus CRT alone in locoregional control. Locoregional control is likely mediated through an increase in cancer cell apoptosis in our model, as shown through *in vitro* and proteomic studies. However, in cases in which locoregional control is improved, as observed in the mEER model, this is associated with reprogramming of the tumor-immune microenvironment toward an immunosuppressive phenotype, which may underlie the lack of survival benefit seen in the clinical trial.

The addition of xevinapant to CRT reversed the CD8^+^ T cell–dependent tumor control in the mEER model because of a reduction in CD8^+^ TIL abundance and effector functionality. Combination treatment led to a trend toward reduced numbers of activated, proliferating, and cytotoxic CD8^+^ T cells and NK cells in the TME compared with CRT alone. *Nr4a3*-Tocky analysis of tumor-filtrating CD8^+^ T cells shows accumulation of the “arrested,” rather than “persistent,” population which have a history of antigen recognition but are no longer reengaging their antigen. Phenotypically, these cells co-express PD-1^hi^ CD38^hi^ markers that represent dysfunctional CD8^+^ T cells because of suboptimal priming which can result in erroneous TCR signaling, unresponsiveness to antigenic restimulation, and αPD-1 resistance (46). Although our data clearly demonstrate the presence of PD-1^hi^ CD38^hi^ CD8^+^ TILs following CRT plus xevinapant, it remains unclear whether their dysfunctional phenotype is cell-intrinsic or a consequence of other suppressive changes in the TME. Further experiments, such as *ex vivo* functional assays following antigen restimulation, could help determine whether dysfunction is reversible and TME-driven or an intrinsic phenotype induced by the treatment. Interestingly, our findings resonate with recent clinical findings in LA-SCCHN. The success of KEYNOTE-689 in the perioperative setting (13) may in part reflect the removal of chronic tumor antigen stimulation through surgery, thereby limiting the development of dysfunctional, exhausted T cells and creating a more favorable immune contexture for effective PD-1 blockade. In contrast, in unresectable disease in which persistent antigen exposure during CRT may reinforce T-cell dysfunction, combining immune checkpoint inhibitors with CRT has thus far failed to provide clinical benefit (14–20). We further investigated the impact of adding αPD-1 to the combination treatment, and the absence of significant enhancement in tumor control or survival suggests that the observed efficacy is likely not T cell–mediated. This is consistent with a previous study that showed that second mitochondria-derived activator of caspase mimetics can inhibit human T-cell proliferation and type 1 cytokine response, strongest with a limiting TCR signal (52).

Our data indicate that the addition of xevinapant to CRT counteracted the CRT-associated upregulation of innate and adaptive immune responses, including pathways related to IFN and cytokine signaling, co-stimulation, chemotaxis, activation, human leukocyte antigen expression, and immune cell abundance, relative to controls. To further assess the translational relevance of our findings, we compared our dataset with two published gene expression signatures associated with immunotherapy responsiveness and prognosis in human SCCHN (47, 48). These signatures include multiple immune-related genes overlapping with those identified in our study. Notably, the addition of xevinapant to CRT resulted in a similarly unfavorable downregulation of these signature genes, particularly in the mEER model, reinforcing the conclusion that xevinapant may impair the immune milieu necessary for effective antitumor responses.

It is known that the type of cancer cell death has immunologic implications. Although whole-proteome analysis of xevinapant in combination with CRT showed a significant increase in several apoptotic markers, there was a significant reduction of RIPK3 levels which is known to mediate necroptosis. Although apoptosis is thought to be immunogenically silent, the activation of RIPK3-mediated necroptosis by therapy has previously been reported to provide long-lasting immunologic protection (53). Additionally, there was a significant increase in acute-phase proteins known to be immunosuppressive with the combination treatment, which further highlights that the addition of xevinapant does not induce a favorable pro-inflammatory TME for TILs to exert an antitumor effect.

This is the first study to show that although xevinapant exerts chemo- and radiosensitizing effects in the irradiated field with increased apoptosis, it simultaneously has immunosuppressive effects. Rather than acting as a direct immunosuppressant, xevinapant may enhance not only the killing of tumor cells but also the killing and/or suppression of immune effector cells within or trafficking through the radiation field, thereby undermining antitumor immunity. This aligns with prior hypotheses from the results of the KEYNOTE-412 trial (20), in which broad radiation fields in LA-SCCHN were speculated to compromise immune cell viability and limit the efficacy of immune checkpoint blockade. Overall, the net effect of CRT plus xevinapant combination seen clinically might be explained by the equivalence of locoregional control (radiosensitization counterbalanced by immunosuppression) but worsening of distant control through reduced systemic antitumor adaptive immunity.

Although our data robustly demonstrate that xevinapant induces immunosuppressive changes in the TME—such as reduced CD8^+^ T-cell abundance and function and altered immune signaling pathways—the direct causal link between these immune alterations and the increased distant failure observed in the TrilynX clinical trial remains to be definitively established. Future studies incorporating longitudinal tracking of immune cell function, metastatic modeling, single-cell sequencing, and correlative analyses in patient-derived samples will be critical to elucidate the mechanisms linking immunomodulation to clinical outcomes.

The efficacy of the combination of xevinapant and CRT could potentially be improved with the addition of drugs that target the DNA damage response to further enhance xevinapant’s radio- and chemosensitizing properties. However, more potent IAP degradation may also increase systemic toxicity and further dampen the antitumor immune response. Although the disappointing results of the TrilynX trial may limit the immediate clinical relevance of IAP inhibitors in LA-SCCHN, our study highlights the broader value of immunocompetent preclinical models for dissecting the immune consequences of combining novel agents with RT/CRT. These models provide critical mechanistic insights that can guide the rational selection and optimization of therapeutic combinations. Notably, the authors of TrilynX themselves have acknowledged that xevinapant may have inadvertently reduced the immune-activating effects of CRT (38). Identifying potential adverse immunosuppressive effects in the preclinical setting could reduce the likelihood of costly negative outcomes in large-scale clinical trials and accelerate the development of more effective immunomodulatory strategies in head and neck oncology.

Given the ongoing disappointing results of phase III trials combining CRT with immunomodulatory agents in LA-SCCHN, our work highlights the urgent need for rigorous, detailed preclinical immune phenotyping—beyond measures of tumor growth delay and short-term survival—to better predict and mitigate adverse impacts on systemic antitumor immunity prior to clinical testing.

## Supplementary Material

Supplementary Data List of anti-mouse monoclonal antibodies used for flow cytometry.

Figure S1 In vitro xevinapant combination with CRT increases MOC1 tumour cell death but not in mEER.

Figure S2 In vivo studies of xevinapant in combination with RT or CRT in the mEER model.

Figure S3 In vivo studies of xevinapant in combination with RT or CRT in the MOC1 model.

Figure S4 Schematic of the Nr4a3-Tocky system.

Figure S5 RNAseq data showing that the addition of xevinapant to CRT does not enhance the immunogenicity of the MOC1 model.

Figure S6 Comparison of our dataset with published gene signatures predicting immunotherapy responsiveness and prognosis in human SCCHN.

Figure S7 Flow cytometry gating strategy for immune profiling.

## Data Availability

Sequencing data have been deposited in the NCBI Gene Expression Omnibus (310 RRID: SCR_005012) under accession number GSE297026. All other data supporting the findings of this study are available in the main manuscript, supplemental files, or from the corresponding author upon reasonable request.

## References

[1] Cancer Research UK . Head and neck cancers statistics. Cancer Research UK. [cited 2024 Mar]. Available from: https://www.cancerresearchuk.org/health-professional/cancer-statistics/statistics-by-cancer-type/head-and-neck-cancers.

[2] Sung H , Ferlay J, Siegel RL, Laversanne M, Soerjomataram I, Jemal A, . Global cancer statistics 2020: GLOBOCAN estimates of incidence and mortality worldwide for 36 cancers in 185 countries. CA Cancer J Clin 2021;71:209–49. 33538338 10.3322/caac.21660

[3] Machiels J-P , René Leemans C, Golusinski W, Grau C, Licitra L, Gregoire V. Squamous cell carcinoma of the oral cavity, larynx, oropharynx and hypopharynx: EHNS-ESMO-ESTRO Clinical Practice Guidelines for diagnosis, treatment and follow-up. Ann Oncol 2020;31:1462–75. 33239190 10.1016/j.annonc.2020.07.011

[4] Ang KK . Multidisciplinary management of locally advanced SCCHN: optimizing treatment outcomes. Oncologist 2008;13:899–910. 18701764 10.1634/theoncologist.2007-0157

[5] Lee Y-G , Kang EJ, Keam B, Choi J-H, Kim J-S, Park KU, . Treatment strategy and outcomes in locally advanced head and neck squamous cell carcinoma: a nationwide retrospective cohort study (KCSG HN13-01). BMC Cancer 2020;20:813. 32854649 10.1186/s12885-020-07297-z PMC7450571

[6] Chow LQM . Head and neck cancer. N Engl J Med 2020;382:60–72. 31893516 10.1056/NEJMra1715715

[7] Grégoire V , Lefebvre J-L, Licitra L, Felip E; EHNS-ESMO-ESTRO Guidelines Working Group. Squamous cell carcinoma of the head and neck: EHNS-ESMO-ESTRO Clinical Practice Guidelines for diagnosis, treatment and follow-up. Ann Oncol 2010;21 Suppl 5:v184–6. 20555077 10.1093/annonc/mdq185

[8] Adelstein DJ , Lavertu P, Saxton JP, Secic M, Wood BG, Wanamaker JR, . Mature results of a phase III randomized trial comparing concurrent chemoradiotherapy with radiation therapy alone in patients with stage III and IV squamous cell carcinoma of the head and neck. Cancer 2000;88:876–83. 10679658 10.1002/(sici)1097-0142(20000215)88:4<876::aid-cncr19>3.0.co;2-y

[9] Suntharalingam M , Haas ML, Van Echo DA, Haddad R, Jacobs MC, Levy S, . Predictors of response and survival after concurrent chemotherapy and radiation for locally advanced squamous cell carcinomas of the head and neck. Cancer 2001;91:548–54. 11169937 10.1002/1097-0142(20010201)91:3<548::aid-cncr1033>3.0.co;2-a

[10] Argiris A , Harrington KJ, Tahara M, Schulten J, Chomette P, Ferreira Castro A, . Evidence-based treatment options in recurrent and/or metastatic squamous cell carcinoma of the head and neck. Front Oncol 2017;7:72. 28536670 10.3389/fonc.2017.00072 PMC5422557

[11] Ionna F , Bossi P, Guida A, Alberti A, Muto P, Salzano G, . Recurrent/metastatic squamous cell carcinoma of the head and neck: a big and intriguing challenge which may be resolved by integrated treatments combining locoregional and systemic therapies. Cancers (Basel) 2021;13:2371. 34069092 10.3390/cancers13102371 PMC8155962

[12] Licitra L , Tahara M, Harrington K, de Mendoza MOH, Guo Y, Aksoy S, . Pembrolizumab with or without lenvatinib as first-line therapy for recurrent or metastatic head and neck squamous cell carcinoma (R/M HNSCC): phase 3 LEAP-010 study. Int J Radiat Oncol Biol Phys 2024;118:e2–3.

[13] Uppaluri R , Haddad RI, Tao Y, Le Tourneau C, Lee NY, Westra W, . Neoadjuvant and adjuvant pembrolizumab in locally advanced head and neck cancer. N Engl J Med 2025;393:37–50. 40532178 10.1056/NEJMoa2415434

[14] Sun XS , Sire C, Tao Y, Martin L, Alfonsi M, Prevost JB, . A phase II randomized trial of pembrolizumab versus cetuximab, concomitant with radiotherapy (RT) in locally advanced (LA) squamous cell carcinoma of the head and neck (SCCHN): first results of the GORTEC 2015-01 “PembroRad” trial. J Clin Oncol 2018;36(Suppl 15):6018.

[15] Tao Y , Aupérin A, Sun X, Sire C, Martin L, Coutte A, . Avelumab-cetuximab-radiotherapy versus standards of care in locally advanced squamous-cell carcinoma of the head and neck: the safety phase of a randomised phase III trial GORTEC 2017-01 (REACH). Eur J Cancer 2020;141:21–9. 33125944 10.1016/j.ejca.2020.09.008

[16] Haddad RW , Wong DJ, Guo Y, Fayette J, Cohen EEW, Kowgier M, . IMvoke010: randomized phase III study of atezolizumab (atezo) as adjuvant monotherapy after definitive therapy of squamous cell carcinoma of the head and neck (SCCHN). Ann Oncol 2019;79(Suppl 13):CT123.

[17] McBride S , Sherman E, Tsai CJ, Baxi S, Aghalar J, Eng J, . Randomized phase II trial of nivolumab with stereotactic body radiotherapy versus nivolumab alone in metastatic head and neck squamous cell carcinoma. J Clin Oncol 2021;39:30–7. 32822275 10.1200/JCO.20.00290 PMC8462641

[18] Yu Y , Lee NY. JAVELIN Head and Neck 100: a Phase III trial of avelumab and chemoradiation for locally advanced head and neck cancer. Future Oncol 2019;15:687–94. 30461306 10.2217/fon-2018-0405 PMC11835014

[19] Lee NY , Ferris RL, Psyrri A, Haddad RI, Tahara M, Bourhis J, . Avelumab plus standard-of-care chemoradiotherapy versus chemoradiotherapy alone in patients with locally advanced squamous cell carcinoma of the head and neck: a randomised, double-blind, placebo-controlled, multicentre, phase 3 trial. Lancet Oncol 2021;22:450–62. 33794205 10.1016/S1470-2045(20)30737-3

[20] Machiels JP , Tao Y, Burtness B, Tahara M, Rischin D, Alves GV, . LBA5 Primary results of the phase III KEYNOTE-412 study: pembrolizumab (pembro) with chemoradiation therapy (CRT) vs placebo plus CRT for locally advanced (LA) head and neck squamous cell carcinoma (HNSCC). Ann Oncol 2022;33(Suppl 7):S1399.

[21] Yang X-H , Feng Z-E, Yan M, Hanada S, Zuo H, Yang C-Z, . XIAP is a predictor of cisplatin-based chemotherapy response and prognosis for patients with advanced head and neck cancer. PLoS One 2012;7:e31601. 22403616 10.1371/journal.pone.0031601 PMC3293890

[22] Tamm I , Kornblau SM, Segall H, Krajewski S, Welsh K, Kitada S, . Expression and prognostic significance of IAP-family genes in human cancers and myeloid leukemias. Clin Cancer Res 2000;6:1796–803. 10815900

[23] Cancer Genome Atlas Network . Comprehensive genomic characterization of head and neck squamous cell carcinomas. Nature 2015;517:576–82. 25631445 10.1038/nature14129 PMC4311405

[24] Fulda S , Vucic D. Targeting IAP proteins for therapeutic intervention in cancer. Nat Rev Drug Discov 2012;11:109–24. 22293567 10.1038/nrd3627

[25] Zheng C , Kabaleeswaran V, Wang Y, Cheng G, Wu H. Crystal structures of the TRAF2: cIAP2 and the TRAF1: TRAF2: cIAP2 complexes: affinity, specificity, and regulation. Mol Cell 2010;38:101–13. 20385093 10.1016/j.molcel.2010.03.009 PMC2855162

[26] Cetraro P , Plaza-Diaz J, MacKenzie A, Abadía-Molina F. A review of the current impact of inhibitors of apoptosis proteins and their repression in cancer. Cancers (Basel) 2022;14:1671. 35406442 10.3390/cancers14071671 PMC8996962

[27] Matzinger O , Viertl D, Tsoutsou P, Kadi L, Rigotti S, Zanna C, . The radiosensitizing activity of the SMAC-mimetic, Debio 1143, is TNFα-mediated in head and neck squamous cell carcinoma. Radiother Oncol 2015;116:495–503. 26096848 10.1016/j.radonc.2015.05.017

[28] Dougan SK , Dougan M. Regulation of innate and adaptive antitumor immunity by IAP antagonists. Immunotherapy 2018;10:787–96. 29807457 10.2217/imt-2017-0185 PMC6462841

[29] Silke J , Meier P. Inhibitor of apoptosis (IAP) proteins-modulators of cell death and inflammation. Cold Spring Harb Perspect Biol 2013;5:a008730. 23378585 10.1101/cshperspect.a008730 PMC3552501

[30] Thibault B , Genre L, Le Naour A, Broca C, Mery E, Vuagniaux G, . DEBIO 1143, an IAP inhibitor, reverses carboplatin resistance in ovarian cancer cells and triggers apoptotic or necroptotic cell death. Sci Rep 2018;8:17862. 30552344 10.1038/s41598-018-35860-z PMC6294826

[31] Serova M , Tijeras-Raballand A, Albert S, Faivre S, Raymond E, Vaslin A, . Abstract 2752: effects of Debio 1143, a novel oral IAP inhibitor, in monotherapy and in combination with platinum drugs in human SCCHN tumor specimens. Cancer Res 2014;74(19 Suppl):2752.

[32] Fleischmann J , Hildebrand LS, Kuhlmann L, Fietkau R, Distel LV. The effect of xevinapant combined with ionizing radiation on HNSCC and normal tissue cells and the impact of xevinapant on its targeted proteins cIAP1 and XIAP. Cells 2023;12:1653. 37371123 10.3390/cells12121653 PMC10297233

[33] Tao Z , McCall NS, Wiedemann N, Vuagniaux G, Yuan Z, Lu B. SMAC mimetic Debio 1143 and ablative radiation therapy synergize to enhance antitumor immunity against lung cancer. Clin Cancer Res 2019;25:1113–24. 30352911 10.1158/1078-0432.CCR-17-3852

[34] Gomez-Roca C , Even C, Le Tourneau C, Basté N, Delord J-P, Sarini J, . Exploratory window-of-opportunity trial to investigate the tumor pharmacokinetics/pharmacodynamics of the IAP antagonist Debio 1143 in patients with head and neck cancer. Clin Transl Sci 2022;15:55–62. 33742767 10.1111/cts.13002 PMC8742634

[35] Sun X-S , Tao Y, Le Tourneau C, Pointreau Y, Sire C, Kaminsky M-C, . Debio 1143 and high-dose cisplatin chemoradiotherapy in high-risk locoregionally advanced squamous cell carcinoma of the head and neck: a double-blind, multicentre, randomised, phase 2 study. Lancet Oncol 2020;21:1173–87. 32758455 10.1016/S1470-2045(20)30327-2

[36] Tao Y , Sun X-S, Pointreau Y, Le Tourneau C, Sire C, Kaminsky M-C, . Extended follow-up of a phase 2 trial of xevinapant plus chemoradiotherapy in high-risk locally advanced squamous cell carcinoma of the head and neck: a randomised clinical trial. Eur J Cancer 2023;183:24–37. 36796234 10.1016/j.ejca.2022.12.015

[37] Merck . Merck provides update on xevinapant program in locally advanced head and neck cancer [Press release]. Merck, a leading science and technology company, today annouced the discontinuation of the Phase III randomized TrilynX study evaluating xevinapant. News. Darmstadt, Germany. 2024 Jun 24. Available from: https://www.merckgroup.com/en/news/xevinapant-update.html.

[38] Bourhis J , Licitra LF, Burtness B, Psyrri A, Haddad R, Harrington K, . Xevinapant or placebo plus platinum-based chemoradiotherapy in unresected locally advanced squamous cell carcinoma of the head and neck (TrilynX): a randomized, phase III study. J Clin Oncol 2025;43:3209–20. 40902136 10.1200/JCO-25-00272 PMC12509437

[39] Bending D , Prieto Martín P, Paduraru A, Ducker C, Marzaganov E, Laviron M, . A timer for analyzing temporally dynamic changes in transcription during differentiation in vivo. J Cell Biol 2018;217:2931–50. 29941474 10.1083/jcb.201711048 PMC6080944

[40] Patro R , Duggal G, Love MI, Irizarry RA, Kingsford C. Salmon provides fast and bias-aware quantification of transcript expression. Nat Methods 2017;14:417–9. 28263959 10.1038/nmeth.4197 PMC5600148

[41] Soneson C , Love MI, Robinson MD. Differential analyses for RNA-seq: transcript-level estimates improve gene-level inferences. F1000Res 2015;4:1521. 26925227 10.12688/f1000research.7563.1 PMC4712774

[42] Love MI , Huber W, Anders S. Moderated estimation of fold change and dispersion for RNA-seq data with DESeq2. Genome Biol 2014;15:550. 25516281 10.1186/s13059-014-0550-8 PMC4302049

[43] Puram SV , Mints M, Pal A, Qi Z, Reeb A, Gelev K, . Cellular states are coupled to genomic and viral heterogeneity in HPV-related oropharyngeal carcinoma. Nat Genet 2023;55:640–50. 37012457 10.1038/s41588-023-01357-3 PMC10191634

[44] Hänzelmann S , Castelo R, Guinney J. GSVA: gene set variation analysis for microarray and RNA-seq data. BMC Bioinformatics 2013;14:7. 23323831 10.1186/1471-2105-14-7 PMC3618321

[45] Xu S , Hu E, Cai Y, Xie Z, Luo X, Zhan L, . Using clusterProfiler to characterize multiomics data. Nat Protoc 2024;19:3292–320. 39019974 10.1038/s41596-024-01020-z

[46] Verma V , Shrimali RK, Ahmad S, Dai W, Wang H, Lu S, . PD-1 blockade in subprimed CD8 cells induces dysfunctional PD-1^+^CD38^hi^ cells and anti-PD-1 resistance. Nat Immunol 2019;20:1231–43. 31358999 10.1038/s41590-019-0441-y PMC7472661

[47] Wang Q , Zhao Y, Wang F, Tan G. A novel immune signature predicts immunotherapy responsiveness and reveals the landscape of the tumor immune microenvironment in head and neck squamous cell carcinoma. Front Genet 2022;13:1051051. 36437964 10.3389/fgene.2022.1051051 PMC9691887

[48] Zhu C , Wu Q, Yang N, Zheng Z, Zhou F, Zhou Y. Immune infiltration characteristics and a gene prognostic signature associated with the immune infiltration in head and neck squamous cell carcinoma. Front Genet 2022;13:848841. 35586567 10.3389/fgene.2022.848841 PMC9108548

[49] Wang F , Huang W, Li A. Serum haptoglobin suppresses T-lymphocyte functions following burns. Chin Med Sci J 1996;11:180–3. 9387406

[50] Yu G , Gao J, Hu W, Hu D, Wang W, Yang S, . ORM1 promotes tumor progression of kidney renal clear cell carcinoma (KIRC) through CALR-mediated apoptosis. Sci Rep 2023;13:15687. 37735575 10.1038/s41598-023-42962-w PMC10514263

[51] Vicuña L , Strochlic DE, Latremoliere A, Bali KK, Simonetti M, Husainie D, . The serine protease inhibitor SerpinA3N attenuates neuropathic pain by inhibiting T cell-derived leukocyte elastase. Nat Med 2015;21:518–23. 25915831 10.1038/nm.3852 PMC4450999

[52] Burton AM , Ligman BR, Kearney CA, Murray SE. SMAC mimetics inhibit human T cell proliferation and fail to augment type 1 cytokine responses. Cell Immunol 2023;384:104674. 36706656 10.1016/j.cellimm.2023.104674 PMC10319349

[53] Mannion J , Gifford V, Bellenie B, Fernando W, Ramos Garcia L, Wilson R, . A RIPK1-specific PROTAC degrader achieves potent antitumor activity by enhancing immunogenic cell death. Immunity 2024;57:1514–32.e15. 38788712 10.1016/j.immuni.2024.04.025 PMC11236506

