# Supplementary material for: Xevinapant plus Chemoradiotherapy Negatively Sculpts the Tumor-Immune Microenvironment in Head and Neck Cancer
Source: Cancer Res Commun. 2025 Nov 27;5(11):2079–91. doi: 10.1158/2767-9764.CRC-25-0604 (PMC12658960; doi:10.1158/2767-9764.CRC-25-0604)
Supplement: Supplementary Data — List of anti-mouse monoclonal antibodies used for flow cytometry. [file crc-25-0604_supplementary_data_suppsd.docx]

# **SUPPLEMENTARY MATERIAL**

The following anti-mouse monoclonal antibodies were used for flow cytometry:

From BD Biosciences:

CD3 (clone: 17A2, BUV563-conjugated, 741319; RRID:AB_2870837),

CD4 (clone: RM4-4, BUV496-conjugated, 741051; RRID:AB_2870666),

CD8 (clone: 53-6.7, BV786-conjugated, 563332; RRID:AB_627207),

CD44 (clone: IM7, BUV395-conjugated, 740215; RRID:AB_3677624),

CD45 (clone: 30-F11, BUV805-conjugated, 748370; RRID:AB_2872789),

CD103 (clone: 2E7, BUV615-conjugated, 751631; RRID:AB_2875624; clone: M290, BUV805-conjugated, 741948; RRID:AB_2871259),

NK1.1 (clone: PK136, BUV737-conjugated, 741715; RRID:AB_630043),

TCRβ (clone: H57-597, BUV737-conjugated, 612821; RRID:AB_2870145).

From BioLegend:

CD4 (clone: RM4-5, PerCP-conjugated, 100537; RRID:AB_394587),

CD25 (clone: PC61, BV650-conjugated, 102037; RRID:AB_2563060),

CD62L (clone: MEL-14, BV510-conjugated, 104441; RRID:AB_2561537; clone: MEL-14, AF700-conjugated, 104426; RRID:AB_3574628),

CD69 (clone: H1.2F3, BV711-conjugated, 104537; RRID:AB_3685235; clone: H1.2F3, PerCP/Cyanine5.5-conjugated, 104521; RRID:AB_940495),

CD38 (clone: 90, PE/Cyanine7-conjugated, 102717; RRID:AB_2275531),

Granzyme B (clone: GB11, AF647-conjugated, 515405; RRID:AB_3166780),

ICOS (clone: C398.4A, BV750-conjugated, 313557; RRID:AB_2876616),

Ki67 (clone: 16A8, FITC-conjugated, 652409; RRID:AB_876936),

NKG2D (clone: CX5, PE/Dazzle594-conjugated, 130213; RRID:AB_2728147),

PD-1 (clone: 29F.1A12, BV605-conjugated, 135219; RRID:AB_2562616; clone: RMP1-30, APC-conjugated, 109111; RRID:AB_10613470),

Perforin (clone: S16009B, PE-conjugated, 154405; RRID:AB_2721640),

TIM-3 (clone: RMT3-23, BV711-conjugated, 119727; RRID:AB_2716208),

TNF-⍺ (clone: MP6-XT22, AF700-conjugated, 506338; RRID:AB_398552).

From Thermo Fisher eBioscience:

FOXP3 (clone: FJK-16s, eFluor450-conjugated, 48-5773-82; RRID:AB_2865134),

T-bet (clone: 4B10, PE/Cyanine7-conjugated, 25-5825-82; RRID:AB_3208274).
