# Supplementary material for: Xevinapant plus Chemoradiotherapy Negatively Sculpts the Tumor-Immune Microenvironment in Head and Neck Cancer
Source: Cancer Res Commun. 2025 Nov 27;5(11):2079–91. doi: 10.1158/2767-9764.CRC-25-0604 (PMC12658960; doi:10.1158/2767-9764.CRC-25-0604)
Supplement: Figure S1 — In vitro xevinapant combination with CRT increases MOC1 tumour cell death but not in mEER. [file crc-25-0604_figure_s1_suppsf1.pptx]

## Slide 1
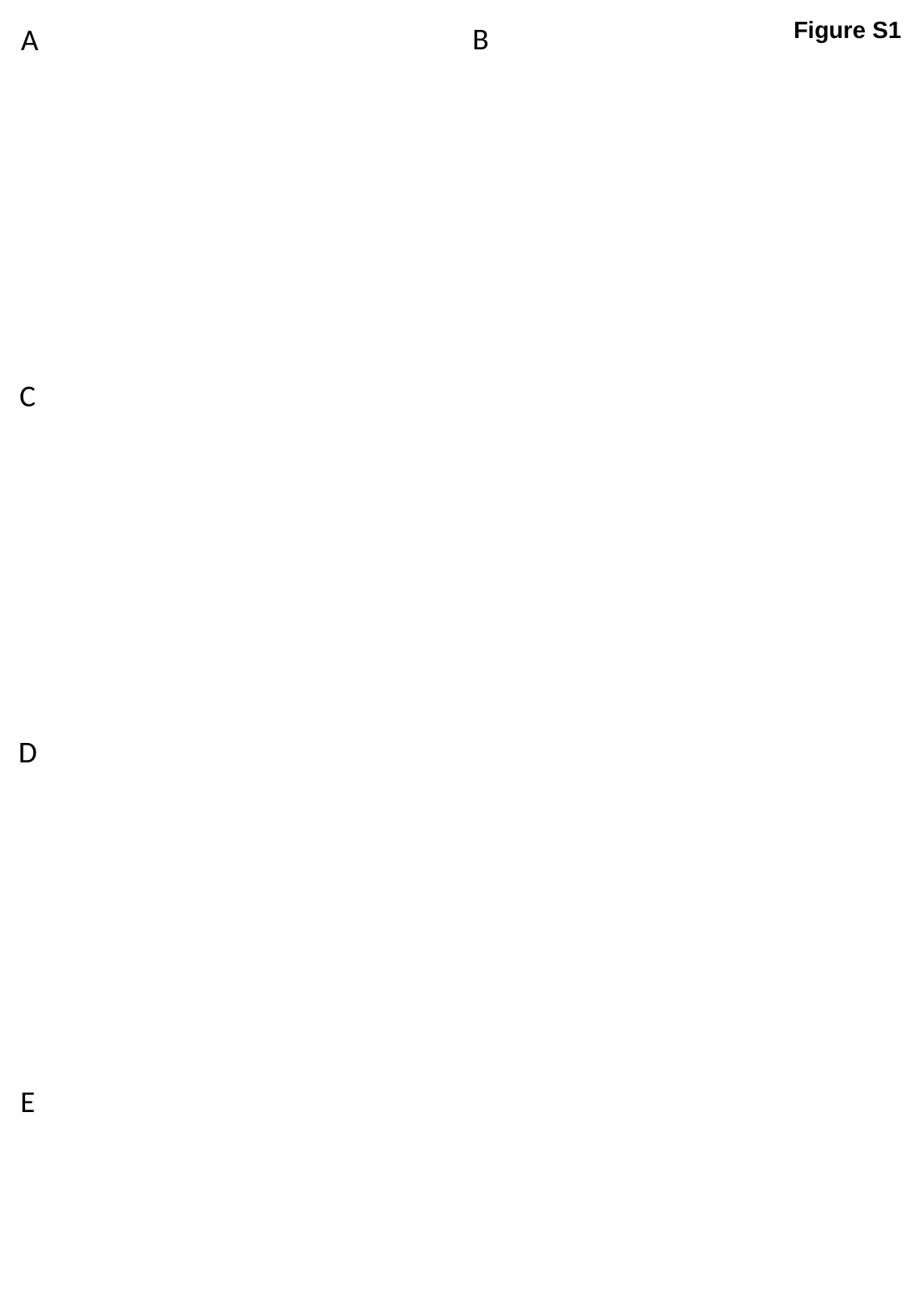

Figure S1
B
A
C
D
E

## Slide 2
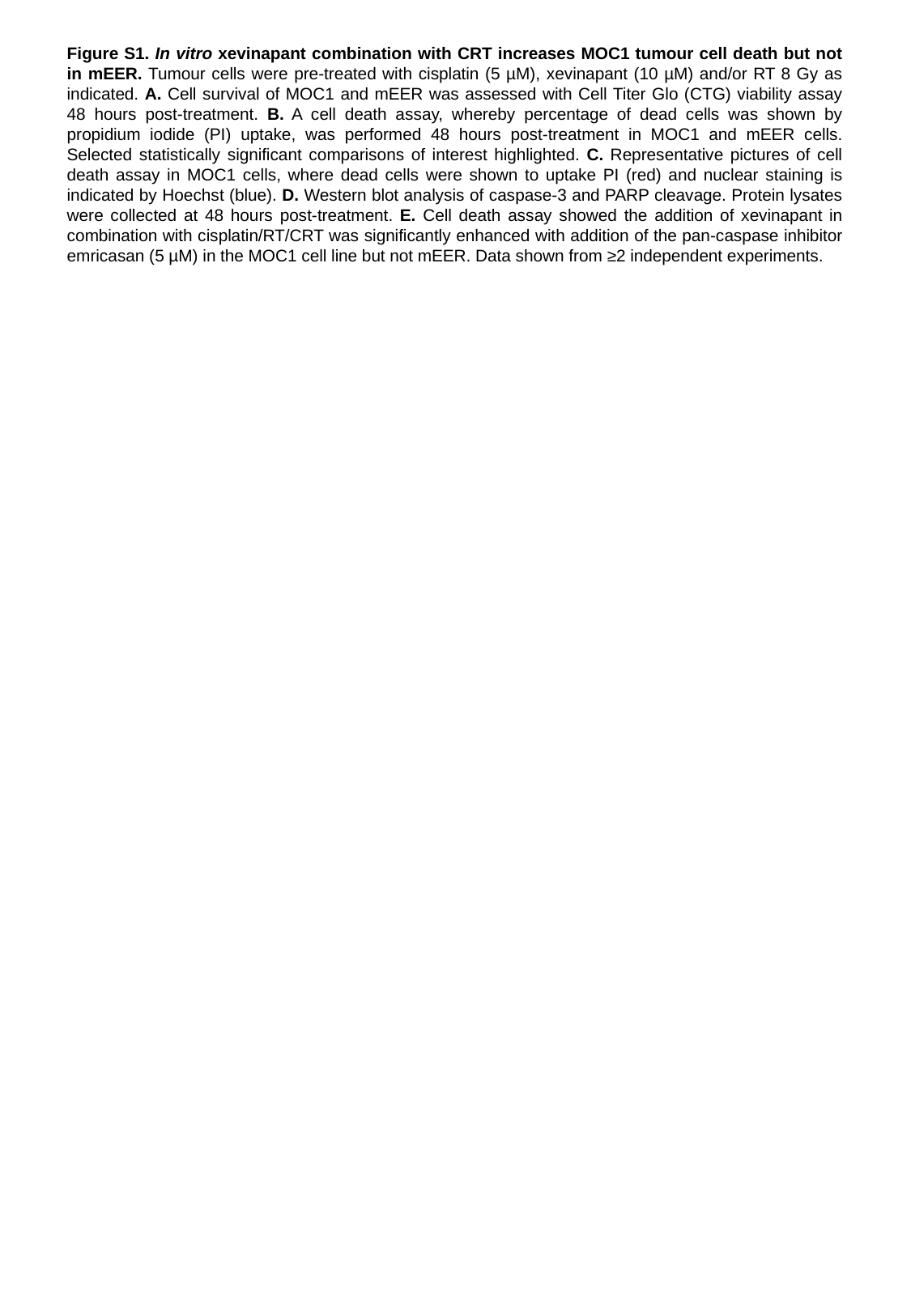

Figure S1. In vitro xevinapant combination with CRT increases MOC1 tumour cell death but not in mEER. Tumour cells were pre-treated with cisplatin (5 µM), xevinapant (10 µM) and/or RT 8 Gy as indicated. A. Cell survival of MOC1 and mEER was assessed with Cell Titer Glo (CTG) viability assay 48 hours post-treatment. B. A cell death assay, whereby percentage of dead cells was shown by propidium iodide (PI) uptake, was performed 48 hours post-treatment in MOC1 and mEER cells. Selected statistically significant comparisons of interest highlighted. C. Representative pictures of cell death assay in MOC1 cells, where dead cells were shown to uptake PI (red) and nuclear staining is indicated by Hoechst (blue). D. Western blot analysis of caspase-3 and PARP cleavage. Protein lysates were collected at 48 hours post-treatment. E. Cell death assay showed the addition of xevinapant in combination with cisplatin/RT/CRT was significantly enhanced with addition of the pan-caspase inhibitor emricasan (5 µM) in the MOC1 cell line but not mEER. Data shown from ≥2 independent experiments.
