# Supplementary material for: Xevinapant plus Chemoradiotherapy Negatively Sculpts the Tumor-Immune Microenvironment in Head and Neck Cancer
Source: Cancer Res Commun. 2025 Nov 27;5(11):2079–91. doi: 10.1158/2767-9764.CRC-25-0604 (PMC12658960; doi:10.1158/2767-9764.CRC-25-0604)
Supplement: Figure S2 — In vivo studies of xevinapant in combination with RT or CRT in the mEER model. [file crc-25-0604_figure_s2_suppsf2.pptx]

## Slide 1
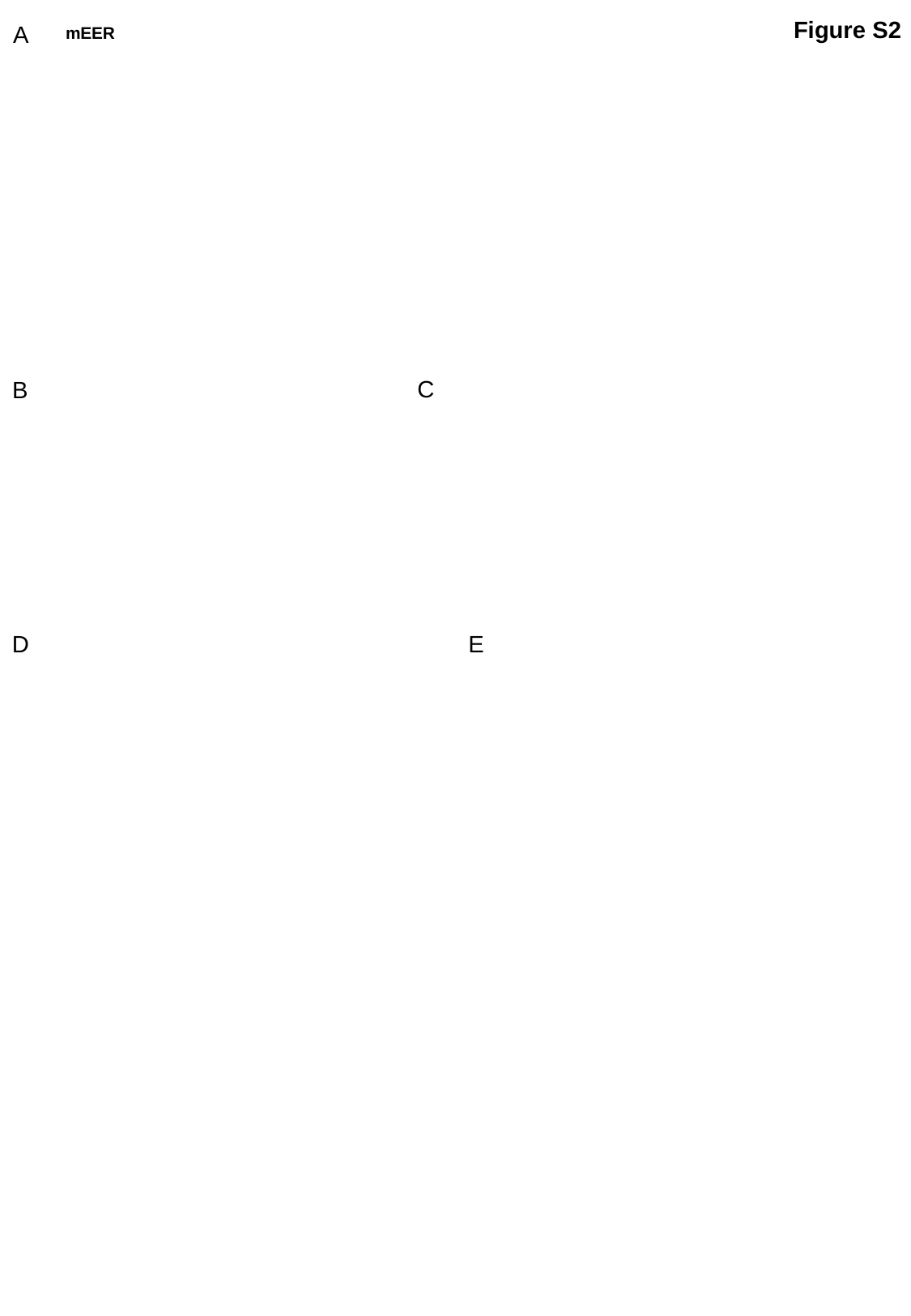

Figure S2
A
mEER
C
B
D
E

## Slide 2
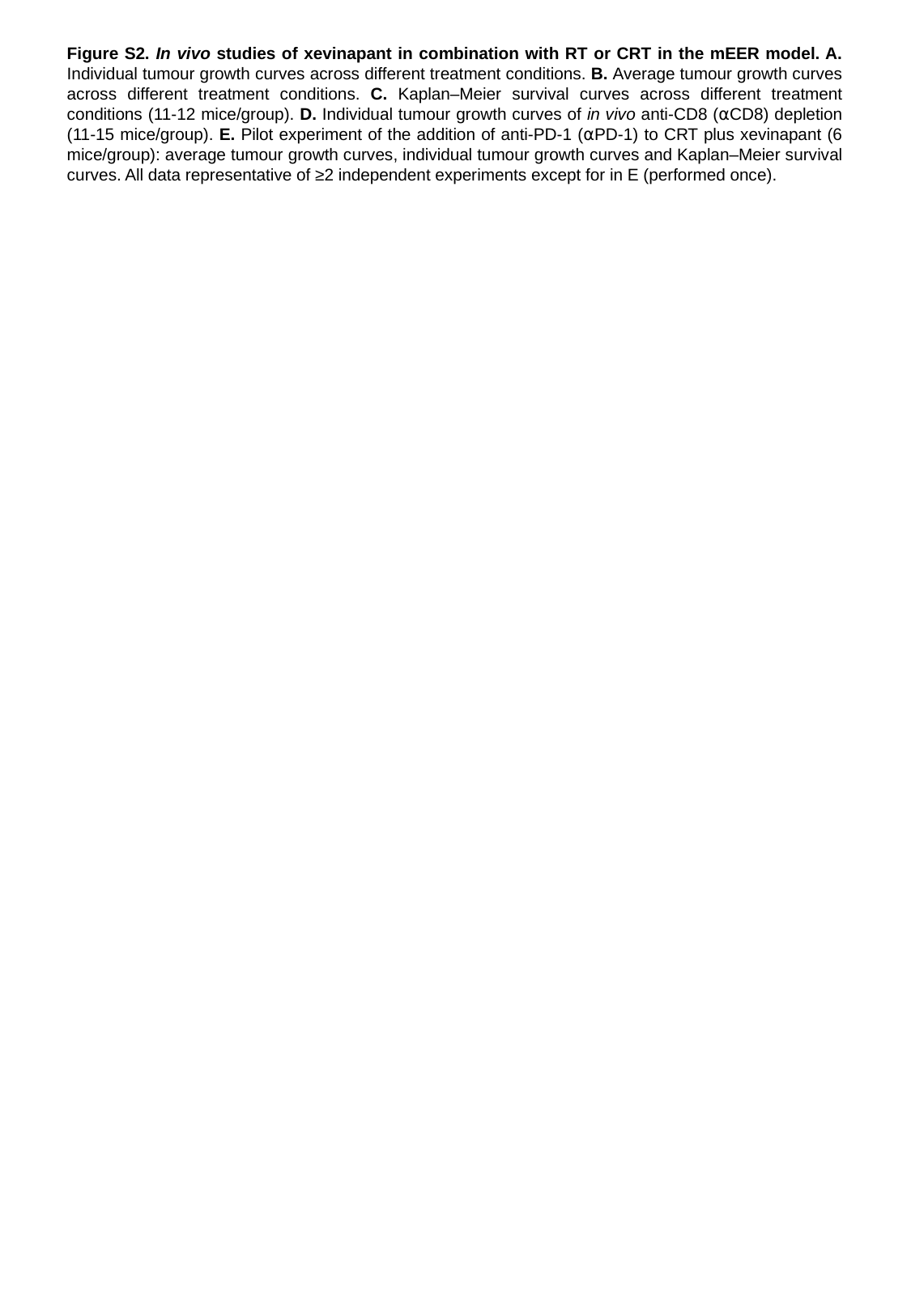

Figure S2. In vivo studies of xevinapant in combination with RT or CRT in the mEER model. A. Individual tumour growth curves across different treatment conditions. B. Average tumour growth curves across different treatment conditions. C. Kaplan–Meier survival curves across different treatment conditions (11-12 mice/group). D. Individual tumour growth curves of in vivo anti-CD8 (⍺CD8) depletion (11-15 mice/group). E. Pilot experiment of the addition of anti-PD-1 (⍺PD-1) to CRT plus xevinapant (6 mice/group): average tumour growth curves, individual tumour growth curves and Kaplan–Meier survival curves. All data representative of ≥2 independent experiments except for in E (performed once).
