# Supplementary material for: Xevinapant plus Chemoradiotherapy Negatively Sculpts the Tumor-Immune Microenvironment in Head and Neck Cancer
Source: Cancer Res Commun. 2025 Nov 27;5(11):2079–91. doi: 10.1158/2767-9764.CRC-25-0604 (PMC12658960; doi:10.1158/2767-9764.CRC-25-0604)
Supplement: Figure S3 — In vivo studies of xevinapant in combination with RT or CRT in the MOC1 model. [file crc-25-0604_figure_s3_suppsf3.pptx]

## Slide 1
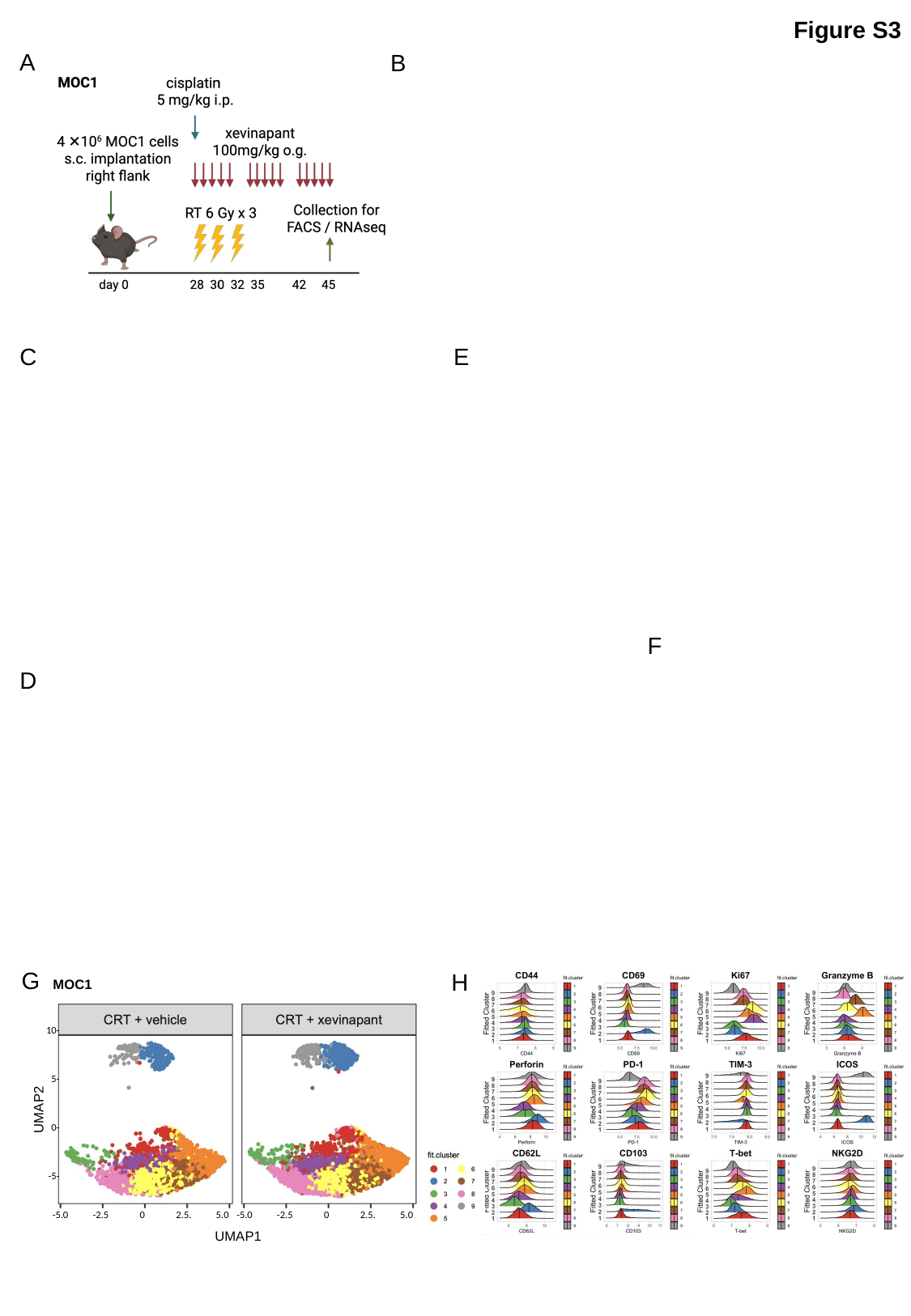

Figure S3
A
B
C
E
F
D
G
H
MOC1
10-
5-
0-
-5-
UMAP2
-5.0 -2.5 0 2.5. 5.0
-5.0 -2.5 0 2.5. 5.0
UMAP1

## Slide 2
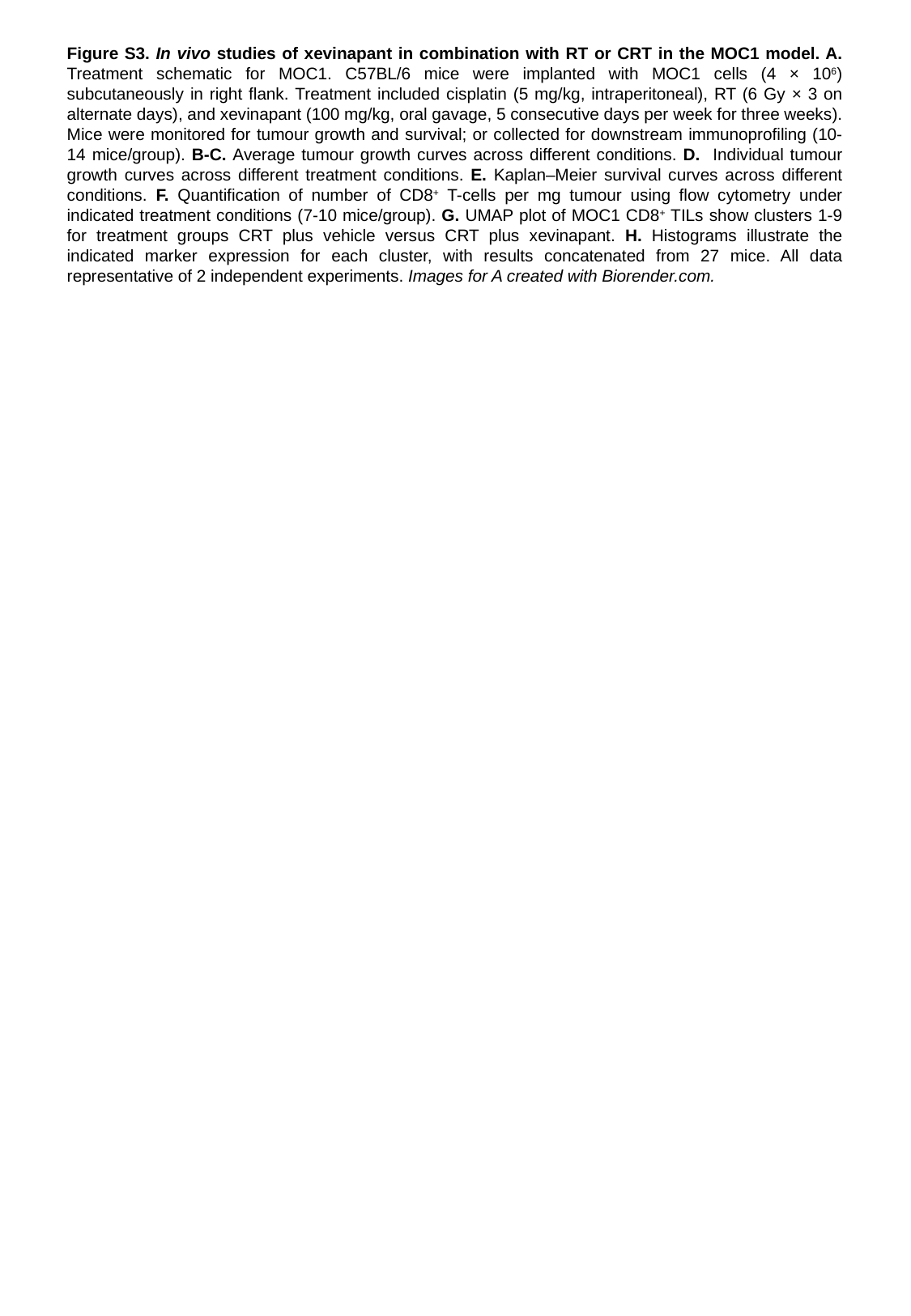

Figure S3. In vivo studies of xevinapant in combination with RT or CRT in the MOC1 model. A. Treatment schematic for MOC1. C57BL/6 mice were implanted with MOC1 cells (4 × 10^6^) subcutaneously in right flank. Treatment included cisplatin (5 mg/kg, intraperitoneal), RT (6 Gy × 3 on alternate days), and xevinapant (100 mg/kg, oral gavage, 5 consecutive days per week for three weeks). Mice were monitored for tumour growth and survival; or collected for downstream immunoprofiling (10-14 mice/group). B-C. Average tumour growth curves across different conditions. D. Individual tumour growth curves across different treatment conditions. E. Kaplan–Meier survival curves across different conditions. F. Quantification of number of CD8+ T-cells per mg tumour using flow cytometry under indicated treatment conditions (7-10 mice/group). G. UMAP plot of MOC1 CD8+ TILs show clusters 1-9 for treatment groups CRT plus vehicle versus CRT plus xevinapant. H. Histograms illustrate the indicated marker expression for each cluster, with results concatenated from 27 mice. All data representative of 2 independent experiments. Images for A created with Biorender.com.
