# Supplementary material for: Xevinapant plus Chemoradiotherapy Negatively Sculpts the Tumor-Immune Microenvironment in Head and Neck Cancer
Source: Cancer Res Commun. 2025 Nov 27;5(11):2079–91. doi: 10.1158/2767-9764.CRC-25-0604 (PMC12658960; doi:10.1158/2767-9764.CRC-25-0604)
Supplement: Figure S4 — Schematic of the Nr4a3-Tocky system. [file crc-25-0604_figure_s4_suppsf4.pptx]

## Slide 1
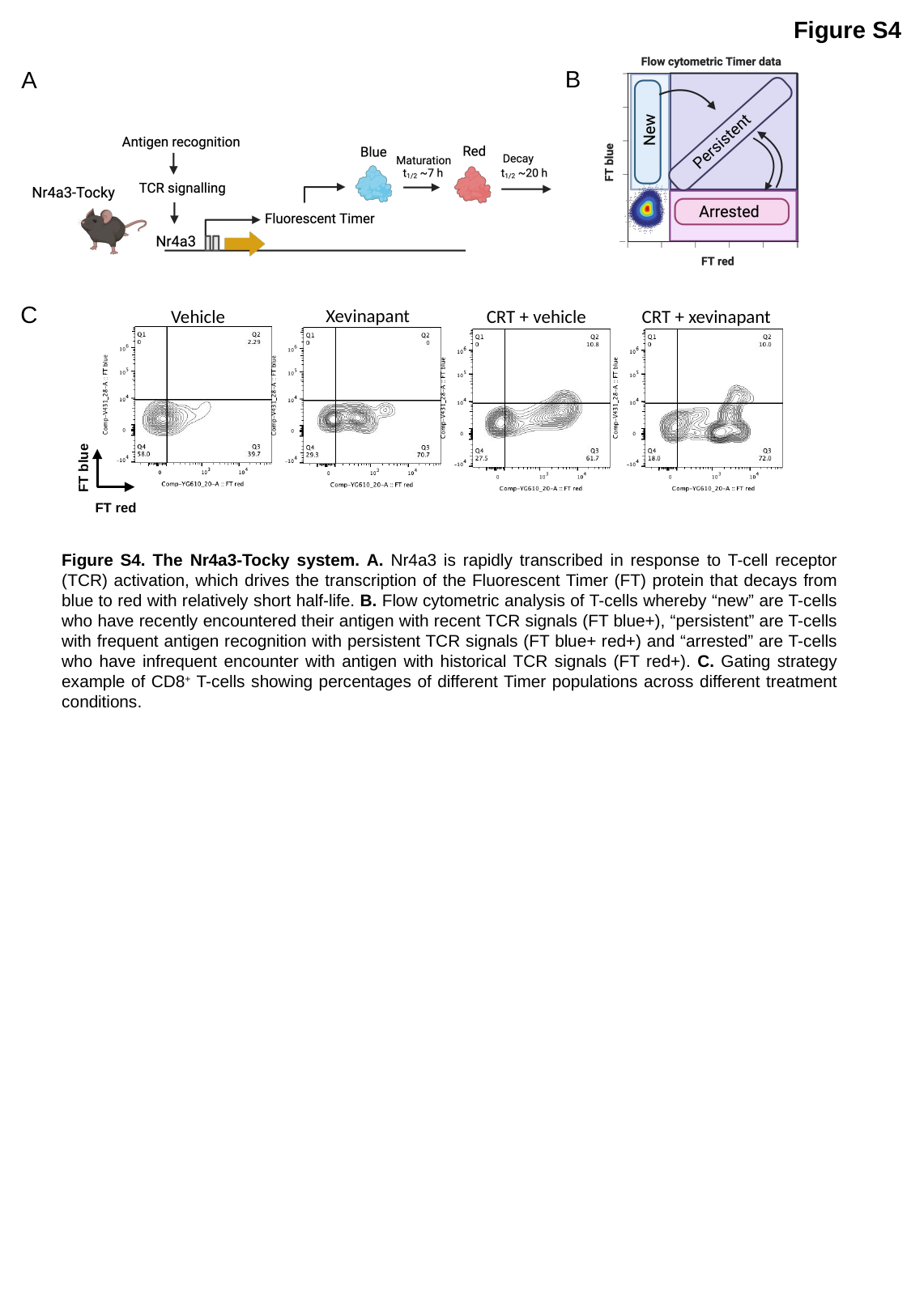

Figure S4
B
A
C
Xevinapant
Vehicle
CRT + vehicle
CRT + xevinapant
FT blue
FT red
Figure S4. The Nr4a3-Tocky system. A. Nr4a3 is rapidly transcribed in response to T-cell receptor (TCR) activation, which drives the transcription of the Fluorescent Timer (FT) protein that decays from blue to red with relatively short half-life. B. Flow cytometric analysis of T-cells whereby “new” are T-cells who have recently encountered their antigen with recent TCR signals (FT blue+), “persistent” are T-cells with frequent antigen recognition with persistent TCR signals (FT blue+ red+) and “arrested” are T-cells who have infrequent encounter with antigen with historical TCR signals (FT red+). C. Gating strategy example of CD8+ T-cells showing percentages of different Timer populations across different treatment conditions.
