# Supplementary material for: Xevinapant plus Chemoradiotherapy Negatively Sculpts the Tumor-Immune Microenvironment in Head and Neck Cancer
Source: Cancer Res Commun. 2025 Nov 27;5(11):2079–91. doi: 10.1158/2767-9764.CRC-25-0604 (PMC12658960; doi:10.1158/2767-9764.CRC-25-0604)
Supplement: Figure S5 — RNAseq data showing that the addition of xevinapant to CRT does not enhance the immunogenicity of the MOC1 model. [file crc-25-0604_figure_s5_suppsf5.pptx]

## Slide 1
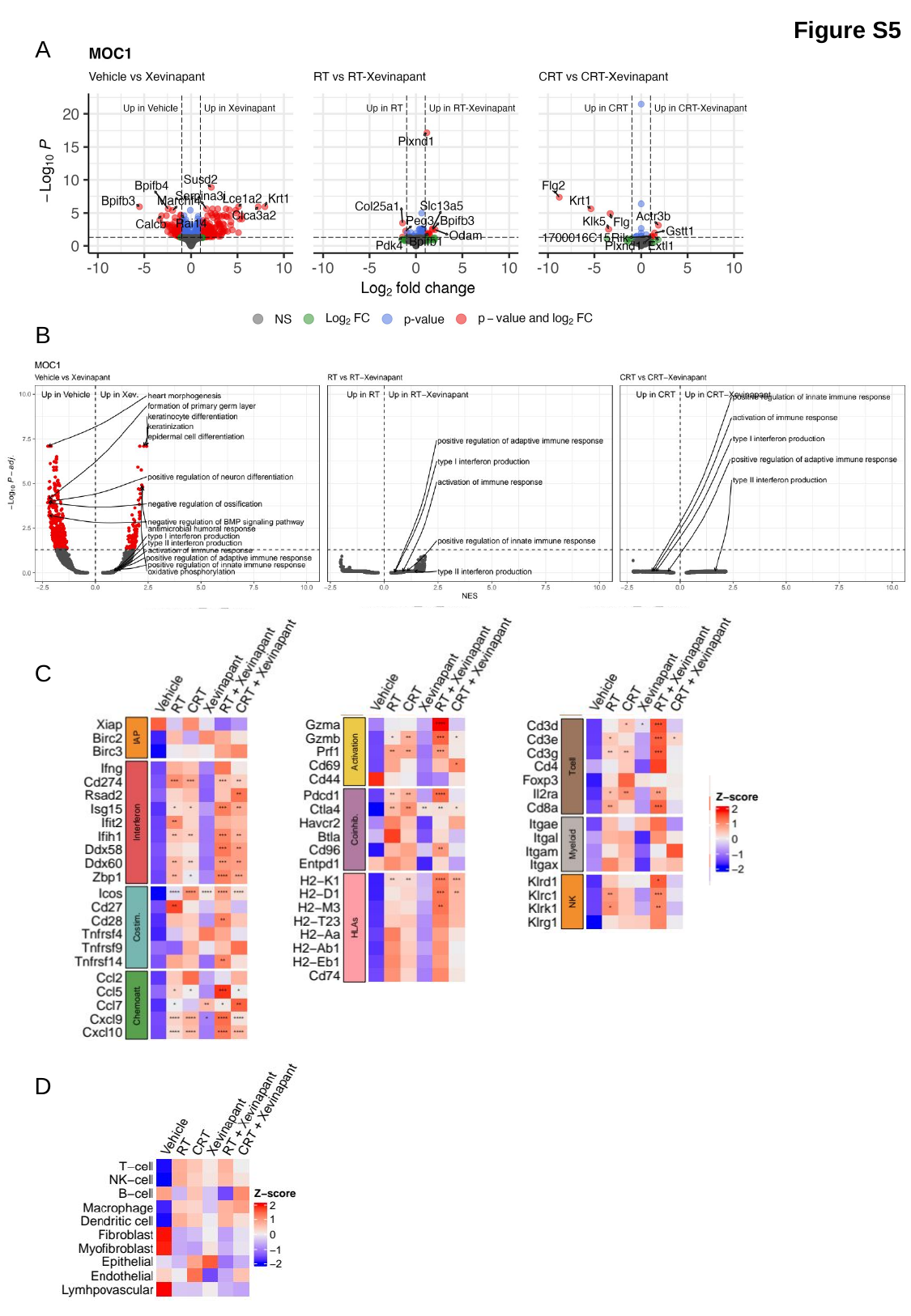

Figure S5
A
B
C
D

## Slide 2
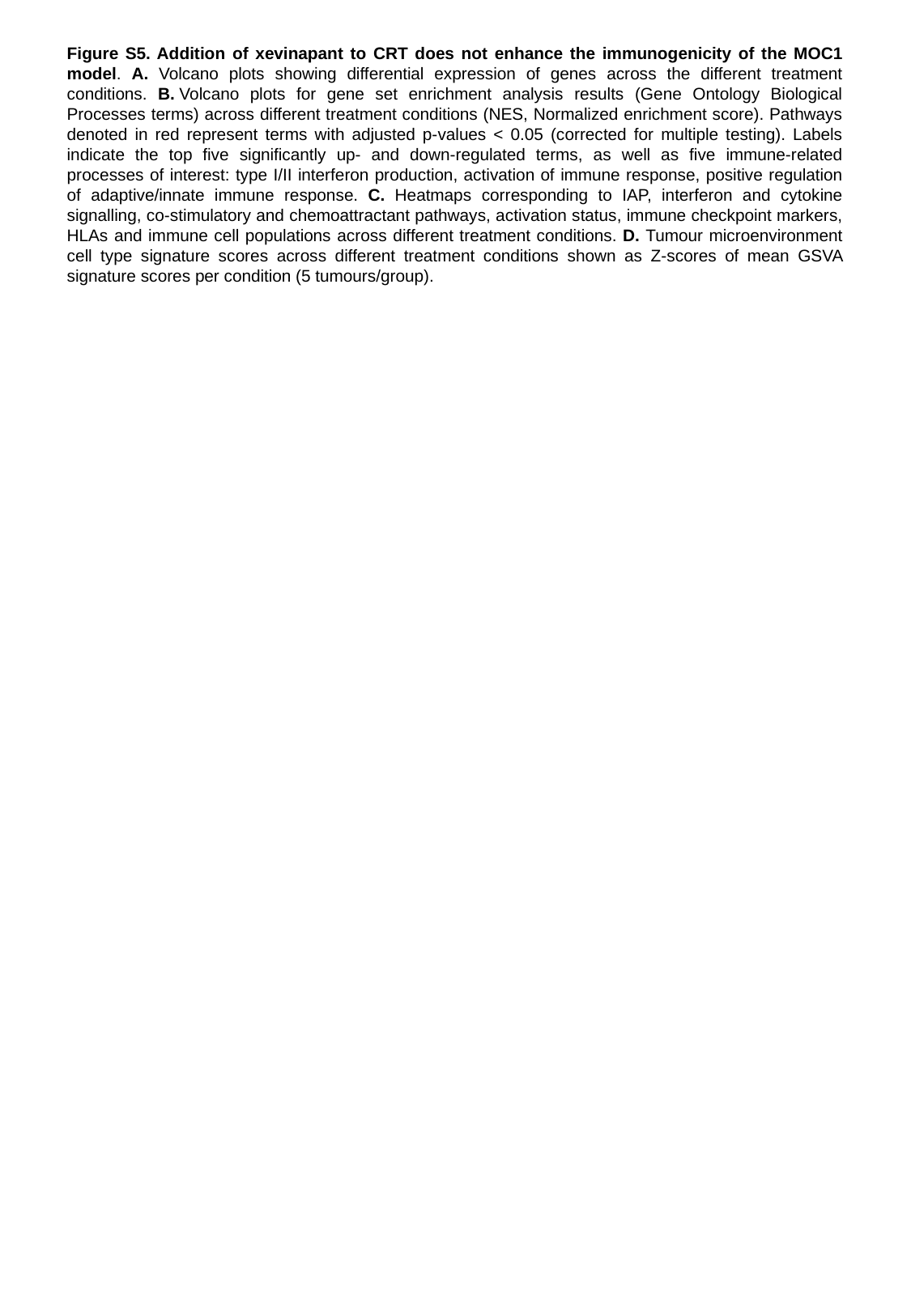

Figure S5. Addition of xevinapant to CRT does not enhance the immunogenicity of the MOC1 model. A. Volcano plots showing differential expression of genes across the different treatment conditions. B. Volcano plots for gene set enrichment analysis results (Gene Ontology Biological Processes terms) across different treatment conditions (NES, Normalized enrichment score). Pathways denoted in red represent terms with adjusted p-values < 0.05 (corrected for multiple testing). Labels indicate the top five significantly up- and down-regulated terms, as well as five immune-related processes of interest: type I/II interferon production, activation of immune response, positive regulation of adaptive/innate immune response. C. Heatmaps corresponding to IAP, interferon and cytokine signalling, co-stimulatory and chemoattractant pathways, activation status, immune checkpoint markers, HLAs and immune cell populations across different treatment conditions. D. Tumour microenvironment cell type signature scores across different treatment conditions shown as Z-scores of mean GSVA signature scores per condition (5 tumours/group).
