# Supplementary material for: Xevinapant plus Chemoradiotherapy Negatively Sculpts the Tumor-Immune Microenvironment in Head and Neck Cancer
Source: Cancer Res Commun. 2025 Nov 27;5(11):2079–91. doi: 10.1158/2767-9764.CRC-25-0604 (PMC12658960; doi:10.1158/2767-9764.CRC-25-0604)
Supplement: Figure S6 — Comparison of our dataset with published gene signatures predicting immunotherapy responsiveness and prognosis in human SCCHN. [file crc-25-0604_figure_s6_suppsf6.pptx]

## Slide 1
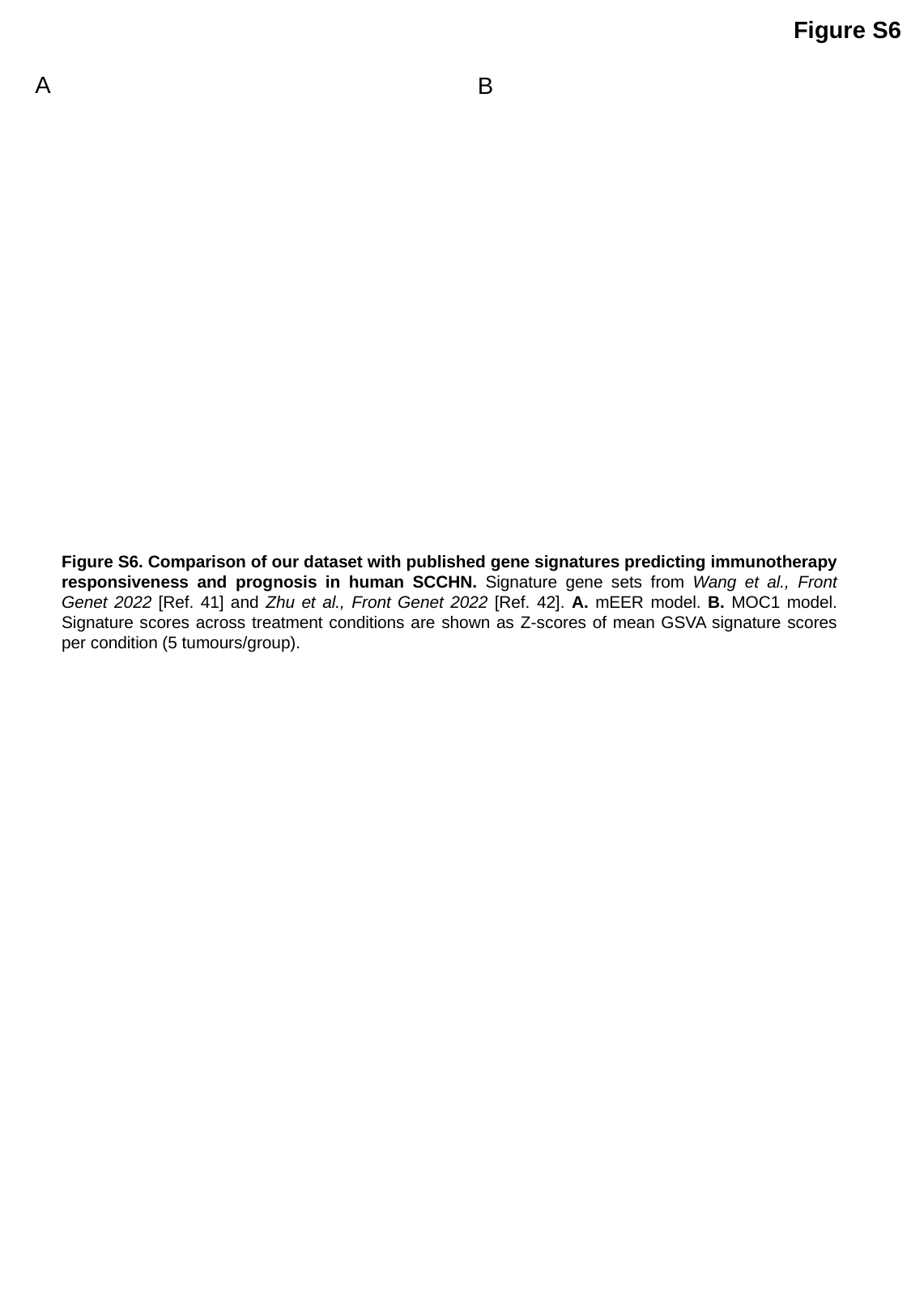

Figure S6
A
B
Figure S6. Comparison of our dataset with published gene signatures predicting immunotherapy responsiveness and prognosis in human SCCHN. Signature gene sets from Wang et al., Front Genet 2022 [Ref. 41] and Zhu et al., Front Genet 2022 [Ref. 42]. A. mEER model. B. MOC1 model. Signature scores across treatment conditions are shown as Z-scores of mean GSVA signature scores per condition (5 tumours/group).
