# Supplementary material for: Xevinapant plus Chemoradiotherapy Negatively Sculpts the Tumor-Immune Microenvironment in Head and Neck Cancer
Source: Cancer Res Commun. 2025 Nov 27;5(11):2079–91. doi: 10.1158/2767-9764.CRC-25-0604 (PMC12658960; doi:10.1158/2767-9764.CRC-25-0604)
Supplement: Figure S7 — Flow cytometry gating strategy for immune profiling. [file crc-25-0604_figure_s7_suppsf7.pptx]

## Slide 1
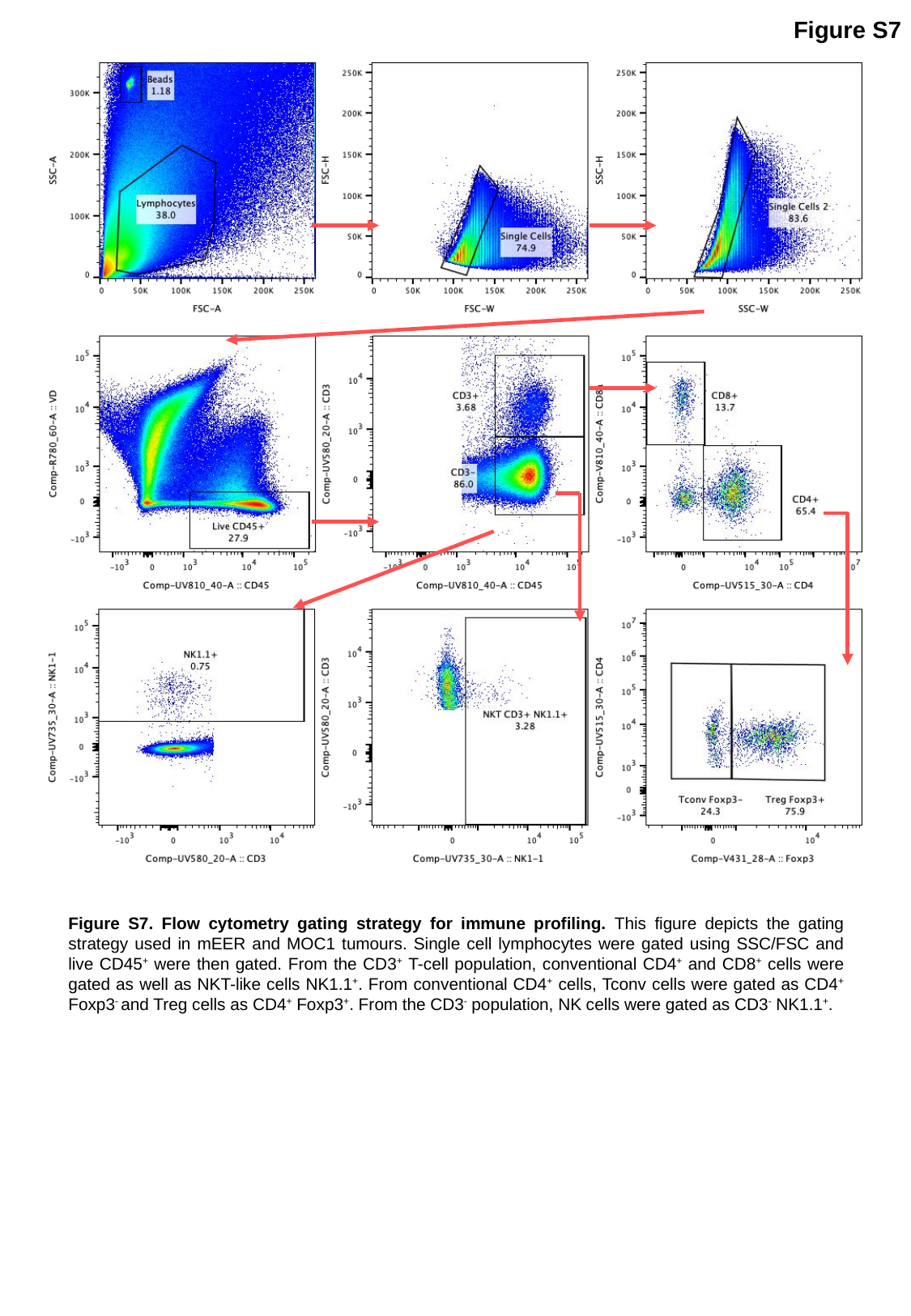

Figure S7
Figure S7. Flow cytometry gating strategy for immune profiling. This figure depicts the gating strategy used in mEER and MOC1 tumours. Single cell lymphocytes were gated using SSC/FSC and live CD45+ were then gated. From the CD3+ T-cell population, conventional CD4+ and CD8+ cells were gated as well as NKT-like cells NK1.1+. From conventional CD4+ cells, Tconv cells were gated as CD4+ Foxp3- and Treg cells as CD4+ Foxp3+. From the CD3- population, NK cells were gated as CD3- NK1.1+.
